# Improving Quality in Nanoparticle-Induced Cytotoxicity Testing by a Tiered Inter-Laboratory Comparison Study

**DOI:** 10.3390/nano10081430

**Published:** 2020-07-22

**Authors:** Inge Nelissen, Andrea Haase, Sergio Anguissola, Louise Rocks, An Jacobs, Hanny Willems, Christian Riebeling, Andreas Luch, Jean-Pascal Piret, Olivier Toussaint, Bénédicte Trouiller, Ghislaine Lacroix, Arno C. Gutleb, Servane Contal, Silvia Diabaté, Carsten Weiss, Tamara Lozano-Fernández, África González-Fernández, Maria Dusinska, Anna Huk, Vicki Stone, Nilesh Kanase, Marek Nocuń, Maciej Stępnik, Stefania Meschini, Maria Grazia Ammendolia, Nastassja Lewinski, Michael Riediker, Marco Venturini, Federico Benetti, Jan Topinka, Tana Brzicova, Silvia Milani, Joachim Rädler, Anna Salvati, Kenneth A. Dawson

**Affiliations:** 1 Health Department, Flemish Institute for Technological Research (VITO), Boeretang 200, 2400 Mol, Belgium; an.jacobs@vito.be (A.J.); hanny.willems@vito.be (H.W.); 2 Department of Chemicals and Product Safety, German Federal Institute for Risk Assessment (BfR), Max-Dohrn-Strasse 8-10, 10589 Berlin, Germany; andrea.haase@bfr.bund.de (A.H.); christian.riebeling@bfr.bund.de (C.R.); andreas.luch@bfr.bund.de (A.L.); 3 Centre for BioNano Interactions, University College Dublin (UCD), Belfield, Dublin 4, Ireland; sergioanguissola@gmail.com (S.A.); Louise.rocks@sfi.ie (L.R.); a.salvati@rug.nl (A.S.); kenneth.a.dawson@cbni.ucd.ie (K.A.D.); 4 Charles River Laboratories, Carrowntreila, Ballina, Co. Mayo, Ireland; 5 Science Foundation Ireland, Three Park Place, Hatch Street Upper, Dublin 2, Ireland; 6 Research Unit in Cellular Biology (URBC), Namur Nanosafety Center (NNC), Namur Research Institute for Life Sciences (NARILIS), University of Namur (UNamur), rue de Bruxelles 61, 5000 Namur, Belgium; jean-pascal.piret@unamur.be; 7 Experimental Toxicology Unit, Institut National de l’Environnement Industriel et des Risques (INERIS), Parc Alata, BP2, 60550 Verneuil-en-Halatte, France; benedicte.trouiller@ineris.fr (B.T.); ghislaine.lacroix@ineris.fr (G.L.); 8 Environmental Research and Innovation (ERIN) Department, Luxembourg Institute of Science and Technology (LIST), 41, rue du Brill, L-4422 Belvaux, Luxembourg; arno.gutleb@list.lu (A.C.G.); servane.contal@list.lu (S.C.); 9 Institute of Toxicology and Genetics, Karlsruhe Institute of Technology (KIT), Hermann-von-Helmholtz-Platz 1, 76344 Eggenstein-Leopoldshafen, Germany; silvia.diabate@kit.edu (S.D.); carsten.weiss@kit.edu (C.W.); 10 Biomedical Research Center (CINBIO), University of Vigo, Campus Lagoas Marcosende, 36310 Vigo, Spain; tlozano@nanoimmunotech.es (T.L.-F.); africa@uvigo.es (Ã.G.-F.); 11 Nanoimmunotech SL, Edificio CITEXVI Fonte das Abelleiras s/n, Campus Universitario de Vigo, 36310 Vigo, Pontevedra, Spain; 12 Instituto de Investigación Sanitaria Galicia Sur (IISGS), Hospital Álvaro Cunqueiro, Estrada Clara Campoamor 341, Babio – Beade, 36312 Vigo, Spain; 13 Health Effects Laboratory, Department of Environmental Chemistry, Norwegian Institute for Air Research (NILU), Instituttveien 18, 2007 Kjeller, Norway; maria.dusinska@nilu.no (M.D.); annahuk8@gmail.com (A.H.); 14 Gentian Diagnostics AS, Bjørnåsveien 5, 1596 Moss, Norway; 15 School of Life Sciences, Heriot-Watt University (HWU), Riccarton Campus, Edinburgh EH14 4AS, UK; v.stone@hw.ac.uk (V.S.); n.kanase@hw.ac.uk (N.K.); 16 Department of Toxicology and Carcinogenesis, Nofer Institute of Occupational Medicine (NIOM), 91-348 Łódź, Poland; mareknocun@gmail.com (M.N.); mstep@imp.lodz.pl (M.S.); 17 SEQme s.r.o., Dlouha 176, 26301 Dobris, Czech Republic; 18 National Center for Drug Research and Evaluation and National Center of Innovative Technologies for Public Health, Istituto Superiore di Sanità (ISS), Viale Regina Elena, 299 Rome, Italy; stefania.meschini@iss.it (S.M.); maria.ammendolia@iss.it (M.G.A.); 19 Institute for Work and Health (IST), University of Lausanne and University of Geneva, Route de la Corniche 2, 1066 Epalinges-Lausanne, Switzerland; nalewinski@vcu.edu (N.L.); michael.riediker@alumni.ethz.ch (M.R.); 20 Department of Chemical and Life Science Engineering, Virginia Commonwealth University, Richmond, VA 23284, USA; 21 Swiss Centre for Occupational and Environmental Health (SCOEH), Binzhofstrasse 87, 8404 Winterthur, Switzerland; 22 School of Materials Science & Engineering, Nanyang Technological University, Block N4.1, Nanyang Avenue, Singapore 639798, Singapore; 23 ECAMRICERT SRL, European Center for the Sustainable Impact of Nanotechnology (ECSIN), Corso Stati Uniti 4, 35127 Padova, Italy; m.venturini@ecamricert.com (M.V.); f.benetti@ecamricert.com (F.B.); 24 Institute of Experimental Medicine (IEM), Czech Academy of Sciences, Videnska 1083, 14220 Prague 4, Czech Republic; jtopinka@iem.cas.cz (J.T.); tana.brzicova@iem.cas.cz (T.B.); 25 Faculty of Safety Engineering, VSB-Technical University of Ostrava, Lumirova 13, 70030 Ostrava-Vyskovice, Czech Republic; 26 Faculty of Physics and Center for NanoScience, Ludwig-Maximilians-Universität, Geshwister-Scholl-Platz 1, 80539 Munich, Germany; s.milani77@googlemail.com (S.M.); raedler@lmu.de (J.R.); 27 Groningen Research Institute of Pharmacy, University of Groningen, A. Deusinglaan 1, 9713AV Groningen, The Netherlands

**Keywords:** nanosafety, cytotoxicity, inter-laboratory comparison, best practice, training

## Abstract

The quality and relevance of nanosafety studies constitute major challenges to ensure their key role as a supporting tool in sustainable innovation, and subsequent competitive economic advantage. However, the number of apparently contradictory and inconclusive research results has increased in the past few years, indicating the need to introduce harmonized protocols and good practices in the nanosafety research community. Therefore, we aimed to evaluate if best-practice training and inter-laboratory comparison (ILC) of performance of the 3-(4,5-dimethylthiazol-2-yl)-5-(3-carboxymethoxyphenyl)-2-(4-sulfophenyl)-2H-tetrazolium (MTS) assay for the cytotoxicity assessment of nanomaterials among 15 European laboratories can improve quality in nanosafety testing. We used two well-described model nanoparticles, 40-nm carboxylated polystyrene (PS-COOH) and 50-nm amino-modified polystyrene (PS-NH2). We followed a tiered approach using well-developed standard operating procedures (SOPs) and sharing the same cells, serum and nanoparticles. We started with determination of the cell growth rate (tier 1), followed by a method transfer phase, in which all laboratories performed the first ILC on the MTS assay (tier 2). Based on the outcome of tier 2 and a survey of laboratory practices, specific training was organized, and the MTS assay SOP was refined. This led to largely improved intra- and inter-laboratory reproducibility in tier 3. In addition, we confirmed that PS-COOH and PS-NH2 are suitable negative and positive control nanoparticles, respectively, to evaluate impact of nanomaterials on cell viability using the MTS assay. Overall, we have demonstrated that the tiered process followed here, with the use of SOPs and representative control nanomaterials, is necessary and makes it possible to achieve good inter-laboratory reproducibility, and therefore high-quality nanotoxicological data.

## 1. Introduction

Within the last 20 years, there has been a tremendous increase in numbers of publications on nanomaterial (NM) toxicity, many of which report inconclusive and controversial results, often apparently conflicting. This has generated a wide debate on the quality and relevance of published papers in nanosafety, including—for instance—a discussion opened up by the *Nature Nanotechnology* journal [1,2], which was followed by several commentaries and other examples [3,4]. A similar discussion about the reliability and reproducibility of experimental data has also been raised for science in general [5,6,7], as it was demonstrated for pre-clinical studies on cancer [8]: using in-house data trying to validate the published results, the authors found that at most only 25% were in line with published data. Efforts to adhere to the biological models as used in the original publications did not improve these results. Conversely, reproducible results were also transferable between models [8]. Largely, discrepancies arise from honest mistakes and flawed statistics, but a recent focus on bad practices and fraud in science has also uncovered instances of the latter [9,10]. Most of the published research has been performed in research laboratories, e.g., at universities, which usually do not adhere to good laboratory practice (GLP) or similar standards. GLP was developed specifically out of an experience of data manipulation and fraud in toxicological contract research [11,12,13]. In regulatory toxicology, adherence to GLP, Organization for Economic Cooperation and Development (OECD) guidelines and International Organization for Standardization (ISO) standards is therefore paramount. Applying similar standards to the existing literature on nanotoxicology by checking publications against a defined set of criteria, such as physico-chemical material characterization or detailed descriptions of the assays applied, including solid statistical data evaluation, resulted in approximately 68% [14] to 90% [15] of the studies being rejected.

Even if it sounds trivial, it has to be stressed that in nanotoxicology all steps from synthesis route to nanoparticle (NP) sample preparation for testing and every step in-between will have an effect on the outcome of the experiments. NMs may become altered throughout all these processes. For biological testing, the dispersion of NMs into media has two crucial aspects, the type of dispersion [16] and the composition of the media [17]. The energy used for the dispersion can, for instance, passivate the surface, influence the agglomeration state and cause dissolution of molecules into ions [18]. Liquid media and biological fluids can influence the same parameters and, importantly, the presence of proteins and other biomolecules leads to the formation of a biocorona on the surface of the NMs [19]. The impact of the corona on NP-cell interactions is demonstrated by the differential cytotoxicity of NPs in the absence or presence of serum, as a general paradigm for all NMs, such as for instance silica [20,21], positively charged NPs [22], carbon nanotubes [23] and graphene oxides [24]. While under realistic exposure conditions in biological fluids the NMs are passivated by the presence of this layer of biomolecules from the surrounding environment, in artificially simplified laboratory conditions, such as serum-free medium, the bare surfaces of NMs can adhere so strongly to the cell surface that they generate damage and other biological processes. Importantly, even the amount and identity of proteins present affects NP outcome on cells [25,26,27], opening up new challenges for determining realistic exposure scenarios. Other unique features of NMs affecting the outcomes of toxicity testing in comparison to standard chemicals include the interference of the NM itself with the testing method: NMs can adsorb and scatter light, interfering with tests based on absorbance, luminescence and fluorescence detection. Furthermore, NMs can also adsorb the reagents used for the tests on their surface, thus causing artefacts that could be misinterpreted for signals [28]. These are just some examples of the many unique features of materials at the nanoscale, which have caused the need for the development of new methods and procedures, as well as specific laboratory practices, in order to be able to generate robust and reproducible data in nanosafety.

These issues are the main reasons why most studies are incomparable [4]. To achieve improved comparability and reproducibility, protocols have to be harmonized and standardized. Moreover, there is a need for the development of alternative testing strategies, as the vast possibilities of engineering NMs would result in a high number of animal studies if current regulatory protocols were to be followed. Animal studies are not only costly, time consuming and ethically fraught; the legislation of cosmetics in the European Union already prohibits this type of testing. Alternative in vitro testing might be a useful tool to prioritize animal testing to NMs of concern. In addition, these methods might be used during R&D to eliminate substances with hazardous properties. Standardization of in vitro procedures requires examining all involved materials and specifying every single step in a protocol. For instance, cell lines have to be identical [29] and free of mycoplasma [30], and even the way how cells are seeded into a multi-well plate has consequences on the test result [31].

In this study, an inter-laboratory comparison (ILC) study among 15 European research laboratories was organized to test how easy it was to generate reproducible data on NP-induced toxicity using standard operating procedures (SOPs), and define processes to enhance the proficiency of nanosafety research laboratories in achieving reliable NM toxicity data. We have employed the in vitro 3-(4,5-dimethylthiazol-2-yl)-5-(3-carboxymethoxyphenyl)-2-(4-sulfophenyl)-2H-tetrazolium (MTS) cytotoxicity assay of the tetrazolium salt reduction-type [32] as a benchmark, which has been addressed before by other ILC consortia consisting of 8 [33], 5 [34] or 6 [35] independent laboratories. Briefly, SOPs were generated by laboratories with previous experience on this assay, as well as in working according to the principles of GLP. The SOP for the MTS assay, including the 96-well plate lay-out and performance criteria, was in agreement with Rösslein et al. [36] and the recently published ISO 19007:2018 standard [37]. Cells, serum and NPs, all from the same batches, were shared among the participating laboratories across Europe, which were enrolled based on the outcome of a first tier on cell culturing proficiency. The second tier was a first MTS inter-laboratory comparison study. Its results, followed by a questionnaire sent to all participants to collect more information on how the procedure was followed, clearly highlighted the need for further optimization of the developed SOP, but also the need of a more precise training in executing this kind of standardized testing. After training of the participants, the third tier consisted of a new round of the MTS assay using the revised SOP. The final results showed a strong reduction in the variability within and across laboratories. Furthermore, our data endorsed the potential of amine-modified polystyrene nanoparticles (PS-NH2) and carboxyl-modified polystyrene nanoparticles (PS-COOH) as positive and negative control nanomaterials, respectively, for cytotoxicity assessment using the MTS assay.

## 2. Materials and Methods

### 2.1. Recruitment of Laboratories

Laboratories involved in nanosafety research across Europe that were (associated) partners of the QualityNano Research Infrastructure consortium were invited to the inter-laboratory comparison study. Before the start of the study, an online questionnaire was sent out to the candidate laboratories to inquire about and evaluate them against criteria mentioned in ISO/IEC 17043:2010 ‘Conformity assessment—general requirements for proficiency testing’ [38], including: experience in biological assessments of NMs or in performing tests similar to the proposed method and/or cell model; the availability of the technical requirements for accommodation, environmental conditions and endpoint measurements; high quality standards for biological testing implemented, such as good cell culture practice or GLP; and trained personnel. In total, 15 laboratories from academia (33.3%), research organizations (60.0%) and industry (6.7%) joined the study. The majority of them were not familiar with the requested high-quality standards that have been developed primarily for regulatory testing or method validation. Moreover, the test performers had varying qualifications (lab technician, PhD student or post-doctoral scientist) and a varied numbers of years of experience in biological testing (from a few months to over 20 years). In most cases, they were not trained in the proposed SOPs.

### 2.2. Choice of Cytotoxicity Test and Materials Used

As mentioned above, we selected the in vitro MTS cytotoxicity assay as a benchmark assay to evaluate and improve laboratories’ proficiency. The CellTiter 96^®^ Aqueous One assay (Promega, Leiden, The Netherlands) used in this study has been identified as superior to other cell viability assays for NP assessment [39]. The assay has originally been developed by Tim Mosmann [32] for the measurement of cell viability using 3-(4,5-dimethylthiazol-2-yl)-2,5-diphenyltetrazolium bromide (MTT). Soluble tetrazolium salts, including 3-(4,5-dimethylthiazol-2-yl)-5-(3-carboxymethoxyphenyl)-2-(4-sulfophenyl)-2H-tetrazolium (MTS), have later been developed, of which the tetrazolium ring is reduced by cellular nicotinamide adenine dinucleotide (phosphate)-dependent oxidoreductase enzymes in the presence of an intermediate electron acceptor (phenazine ethosulfate), to form a formazan derivative that is quantified in a spectrophotometer. The product therefore reflects the metabolic activity and by extension viability of cells, and, hence, can be used to determine a toxic dose of a substance. Information on the cytotoxicity of a substance in alternative methods using cell culture is a crucial first screening step to any more detailed investigation, or as part of a safe-by-design approach. Test methods to assess the in vitro cytotoxicity of medical devices have been described in ISO 10993-5:2009 [40]. A standard specifically dedicated to nanomaterials using the MTS assay as an in vitro cytotoxicity assay has more recently been published [37].

The human A549 alveolar epithelial cell line was chosen as a cell type representing the respiratory route of exposure and a major site of deposition of small nanoparticles [41,42]. Moreover, this cell model is widely used in nanosafety laboratories and easy to maintain, and therefore suitable for standardization among different laboratories.

Alternative methods for toxicity testing require substances with known potential, either positive or negative control substances, which are crucial as quality controls in SOPs. So far, several NM controls have been suggested for in vitro nanoparticle toxicity studies, including tungsten carbide-cobalt [43] as positive, and barium sulfate [44] and carboxylated nanodiamonds [45] as negative controls. As is true for chemicals, any NM that would be used as a control for a specific assay needs to be thoroughly characterized with respect to its physico-chemical properties, and its performance in this assay also needs to be well described. However, for NMs special care has to be taken, because of possible batch-to-batch variation, contamination and long-term stability [46]. In our study, we have selected 50-nm amine-modified polystyrene NPs (PS-NH2) and 40-nm carboxyl-modified polystyrene NPs (PS-COOH), which are known to form stable dispersions when diluted in cell culture medium [47,48,49]. Furthermore, their impact on cells has been characterized in detail [22,26,48,50,51,52]. Thus, they constituted ideal candidates as starting materials for this ILC study.

### 2.3. Standardization Procedures and SOP Development

Standard operating procedures for cell culturing and cell growth rate determination (tier 1), and cytotoxicity assessment using the MTS assay (tier 2 and 3) were adopted from existing protocols, and adapted to implement the spirit of Good Cell Culture Practice [53] and GLP [13], and to be in line with the ISO 19007:2018(E) standard [37]. Forms for detailed registration of performance of the protocol steps were prepared and filled in by the partner laboratories, to make it possible to formulate corrective actions in case of deviations in a laboratory’s results. For data analysis and reporting, spreadsheet (Microsoft Excel) templates and web forms were prepared and distributed to the laboratories. These enabled automated calculations and immediate evaluation of compliance with the test acceptance criteria as formulated in the SOPs. After a first ILC on the MTS assay (tier 2) with the developed SOPs, further changes were made to the MTS assay SOP, and forms based on the feedback from the participating laboratories through an online questionnaire. The optimized SOPs and the spreadsheet templates are available in Supplementary Materials.

To enhance standardization in cell growth and test performance, all laboratories used the most critical materials, such as fetal bovine serum and test NPs from a centrally held stock prepared at one location. Fresh aliquots were shipped to the laboratories prior to the start of the studies. In addition, identical frozen cell stocks of the human A549 alveolar epithelial cell line were obtained by the participating laboratories from a central laboratory, which purchased a parent cell line (ATCC, CCL-185, passage number 82) and subcultured the cells up to a master cell bank (passage number 88). A harmonized SOP for thawing, freezing and subculturing of the A549 cell line, and testing for mycoplasma contamination according to an in-house protocol as an essential quality control before freezing the cells, was developed and used by the study partners to generate their own working cell bank. Other critical reagents, such as cell culturing reagents, MTS reagent and staurosporine were used from the same supplier by the laboratories.

### 2.4. Nanoparticles and Chemical Control

PS-NH2 (50 nm) and fluorescently labelled PS-COOH (40 nm) were purchased from Bangs Laboratories Inc. (Fishers, IN, USA; catalogue number PA02N) and Molecular Probes (ThermoFisher Scientific, Bio-Sciences, Dublin, Ireland; catalogue number F8795), respectively. Aliquots of diluted NPs (10 mg/mL) in milliQ water (resistivity of 18.2 mΩ.cm at 25 °C) were prepared centrally, distributed among all participants, and stored at 4 °C. Dispersions of the NPs of 100 µg/mL in complete cell culture medium (CCM) containing minimal essential medium with GlutaMAX™ (Gibco^®^, Life Technologies, Paisley, UK), supplemented with 10% (*v*/*v*) non-heat inactivated fetal bovine serum (FBS; Gibco^®^, Life Technologies, Paisley, UK), 100 U/mL penicillin and 100 µg/mL streptomycin (Gibco^®^, Invitrogen, Paisley, UK) were prepared by each laboratory. This was done by pipetting 20 µL of the NP stock suspensions in 1980 µL medium and mixing on a vortex for 30 s.

Staurosporine (Proteinkinase, Biaffin GmbH & Co., KG, Kassel, Germany; catalogue number PKI-STSP-001) was used as a positive chemical control to serve as an internal control of the biological cell response and MTS assay performance. As this compound was observed to become instable and lose its activity during transport and storage when prepared as solution, all laboratories were asked to purchase their own lot of lyophilized powder from the same company and with the same batch number. A stock solution of 1 mM staurosporine was prepared by dissolving the powder in 214 µL dimethyl sulfoxide (DMSO) and mixing on a vortex, followed by immediate further dilution to 2140 µL DMSO. Aliquots of 50 µL stock solution were stored at −80 °C without loss of activity for at least 6 months, and a fresh tube was thawed for each experiment. Solutions of 1000 nM staurosporine were prepared in complete CCM containing 1% milliQ water to ensure identical vehicle to the NPs. Details on the test item preparations are described in the SOP for the MTS cytotoxicity assay (Supplementary Materials).

### 2.5. Cell Culture and Exposure

Human A549 alveolar epithelial cells were maintained in complete CCM without antibiotics. The cells were subcultured every 3–4 days when the cell monolayer reached 70–80% confluence, with medium renewal after 2 days. Cells were used for testing up to passage number 20, to ensure equally low passage numbers among experiments and laboratories. Cells were used at >90% viability. Details are in the SOP for A549 cell culturing in Supplementary Materials.

Cell growth curves were obtained as detailed in the SOP for assessment of A549 cell growth rate and viability (Supplementary Materials) by determining the cell number and viability by trypan blue exclusion at 24 h, 48 h and 72 h after seeding.

Assessment of NP-induced cytotoxicity by the MTS assay was performed in 96-well plates, in which 200 µL of cell suspension were seeded per well, followed by 24 h incubation at 37 °C, 5% CO_2_ to allow for cell adhesion prior to exposure to the test items. The dosing plate layout, shown in Figure S1, contained three replicate series of 6 concentrations of either the PS-NH2 or PS-COOH NPs (0; 1; 10; 25; 50 and 100 µg/mL) and 6 concentrations of staurosporine (0; 62.5; 125; 250; 500; and 1000 nM) as a positive chemical control, in addition to six replicate wells for each of 4 assay controls, according to Rösslein et al. [36]. Triplicate dilution series of the NPs and staurosporine were each started from a separate preparation of the highest test concentration, in order to estimate within-laboratory variability in the preparation. Dose series of the test items were obtained by serial dilutions in complete CCM supplemented with antibiotics, and containing 1% milliQ water to ensure identical vehicle in each well. Assay controls consisted of untreated cells in complete CCM with 1% milliQ water, blank wells (no cells) containing complete CCM with 1% milliQ water, and blank wells (no cells) containing the highest test concentrations of the NPs and staurosporine to control for potential interference with the assay read-out measurement. Cells were exposed by the removal of the medium from each well and transfer of 100 µL of the chemical and NP doses from the dosing plate, and, again, incubated for 24 h. All details and plate layouts can be found in the SOP for the MTS cytotoxicity assay (Supplementary Materials).

### 2.6. MTS Assay

At the end of the incubation of cells with the NPs and staurosporine doses, the medium was removed from each well and replaced with 150 µL of diluted CellTiter 96^®^ AQueous One Solution Reagent containing MTS and the electron coupling reagent phenazine ethosulfate (Promega, Leiden, The Netherlands). Cell plates were incubated at 37 °C and 5% CO_2_ for 1 h, to allow for bioreduction of the tetrazolium compound to a colored, soluble formazan product. The absorbance of the product at 490 nm, which is directly proportional to the number of living cells in culture was recorded using a spectrophotometer. Measurements were performed in the presence of the cells, and—after SOP optimization—after transfer of 100 µL of the colored reagent from the cell plate into a new 96-well plate, to ensure absorbance read-out in the linear dynamic range relevant to the Lambert-Beer law.

### 2.7. Statistical Data Analysis and Proficiency Testing

At least 3 independent runs executed on different days using cell cultures of different passage numbers were performed for cell growth rate determination (tier 1) and the MTS assay (tier 2 and 3). An initial analysis of the raw data was done by the individual laboratories using a spreadsheet calculation template enabling immediate evaluation of compliance with the test acceptance criteria mentioned in the SOPs, as well as automated data analysis and plotting (Supplementary Materials). Only data sets from fully acceptable tests were considered valid to be included in the final statistical analysis.

In the determination of cell growth rate, the total cell counts and percentages of cell viability were calculated, based on the live and dead cell numbers. A curve presenting the live cell counts at different growth times (24, 48 and 72 h) was used for exponential fitting (Figure S2). The prefactor of the exponential power from the resulting equation indicated the relative growth rate (doublings per hour), and was used to calculate the cell doubling time (hours).

In a run of the MTS assay, the percentage cell survival was calculated as the fraction of cells that remained viable after treatment, by subtracting the average background absorbance of medium blank wells (Figure S1, column 7) from each raw absorbance value, and normalizing the resulting values to the average absorbance of untreated cells. The triplicate values from a single dose were used to calculate a mean value and standard deviation (SD).

The statistical analysis of the MTS assay data from all laboratories was automated using in-house programming in R software [54]. Fitted sigmoidal curves with the upper limit fixed to 100 and lower limit to 0 were generated per run based on the dose-response data, and used to calculate the effective concentrations causing 30% (EC30, for staurosporine) or 50% (EC50, for PS-NH2) inhibition of cell viability. For curve fitting the R package drc was used [55] and the four-parameter logistic model with parameters b, c, d, e:

f (*x*, (b, c, d, e)) = c + (d − c)/(1 + exp[b (log(*x*) − log(e))] (1)

Parameter e corresponds to the EC30 or EC50, whereas parameter b denotes the relative slope around e. The logistic function is symmetric around e. Examples of resulting dose-response curves per run of two different laboratories are shown in Figure S3.

For proficiency testing by inter-laboratory comparisons, statistical methods according to ISO 13258:2005(E) were followed in this study. More specifically, a robust statistical approach described in algorithm A [56] was applied to calculate robust values of the mean and SD of cell doubling times, % cell survival per dose, and EC30 (or EC50) from at least 3 independent runs reported by the individual participants in a round of the proficiency testing scheme. Next, the robust overall mean and SD from the data of all laboratories were calculated, starting from the robust mean data per lab using the algorithm A. Additionally, for the MTS data, based on the robust within-laboratory SD for each dose or EC30 (or EC50), a robust overall SD* was calculated using algorithm S [56], which yields a robust pooled value of the SD values, to which it is applied. Intra- and inter-laboratory biases were interpreted on the basis of overall mean and SD values derived from all laboratories. We concluded that a laboratory was proficient if the robust within-laboratory mean value did not exceed the overall mean with more than 2-fold the overall SD. Similarly, if the SD values of one laboratory were within the range of twice the overall SD*, then the laboratory bias at the individual dose or EC30 (or EC50) level was considered acceptable.

Finally, a coefficient of variation (CV) was calculated to evaluate the reproducibility of assay performance as: (robust SD/robust mean) × 100%. Variability of biological test results within or between laboratories was considered acceptable if CV < 30%.

## 3. Results

### 3.1. Determination of Cell Growth Rate (Tier 1)

**Figure 1:**
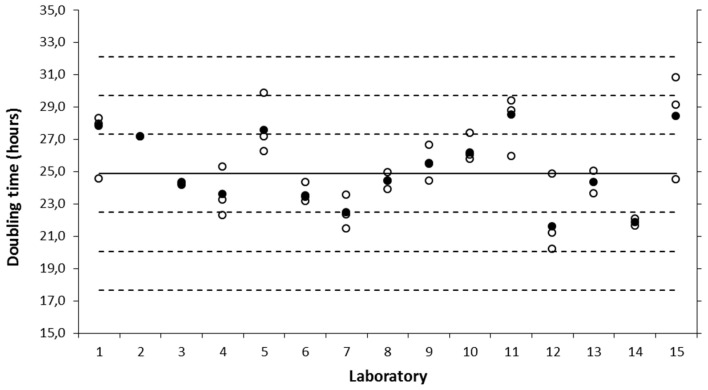
Inter-laboratory evaluation of growth rate of the A549 cell line (tier 1). Three (some 2) independent runs (indicated with open circles) were performed in each laboratory to determine the cell doubling time. The mean of each lab (filled circles), and overall mean (N = 15; black solid line) with 1-, 2- or 3-fold of the overall standard deviation (SD) (black dotted lines) are indicated.

In a first tier of the study, an SOP on A549 cell culturing and an SOP on determination of cell growth rate and viability (Supplementary Materials) were used by the 15 participating laboratories, to assess variability in the cell growth characteristics. Cell cultures for independent runs were started from independent cell vials of the working cell bank and tested for the absence of mycoplasma infection using in-house procedures. Relative cell viability was tested by trypan blue exclusion and was observed to fulfil the acceptance criterion (>90%) mentioned in the cell culturing SOP in the different laboratories. The mean cell doubling time derived from individual cell growth curves of all laboratories was 24.9 ± 2.4 h, and showed good agreement within and between the participating laboratories. Each laboratory produced at least two independent measurements, resulting in a mean doubling time within the boundaries of the overall mean ± 2SD, which indicated that they were all proficient in cell culturing (Figure 1). The largest variability was observed within laboratory 15, which obtained a mean cell doubling time of 28.4 ± 3.2 h.

### 3.2. Assessment of Laboratories’ Inherent Proficiency in Performing the In Vitro MTS Assay (Tier 2)

After benchmarking and confirming the proficiency of the laboratories in their cell culturing performance, they were enrolled in a second tier of the inter-laboratory comparison study involving the transfer of the SOP on the MTS assay. Here, we aimed to evaluate the inherent proficiency of each participating laboratory, and identify critical phases in the SOP that introduce bias in testing, to allow further optimization of the SOP. For this study tier, the laboratories performed independent runs using cells of different passage number from the same working cell bank vial. Results on cytotoxicity assessment of the positive chemical control staurosporine showed that eight out of 15 laboratories reported on at least three independent and valid runs, based on assessment against the test acceptance criteria mentioned in the SOP. For the tests with PS-COOH and PS-NH2, however, only five and four out of 15 participating laboratories, respectively, were able to generate at least three valid runs (Table 1). The most frequent reason for this low rate of valid runs was a deviation of more than 15% in the absorbance values from triplicate cultures treated with zero dose of the test item (staurosporine or NPs; Figure S1, row B), as compared to untreated cultures (Figure S1, column 6). This can be attributed to the differences in seeded cell numbers or cell densities in the respective wells of the multi-well plate, which may have multiple causes, such as the poor resuspension of cells while seeding, inaccurate pipetting volumes, wrong pipetting technique, etc. Furthermore, about half of the laboratories observed interference of the PS-NH2 with the absorbance read-out for more than 15% compared to blank wells containing no NPs, which was set as a limit for acceptance of the test. More detailed data investigation revealed that this was mainly due to high variability (CV >30%) between replicate wells of the NP blank within these laboratories.

**Figure 2:**
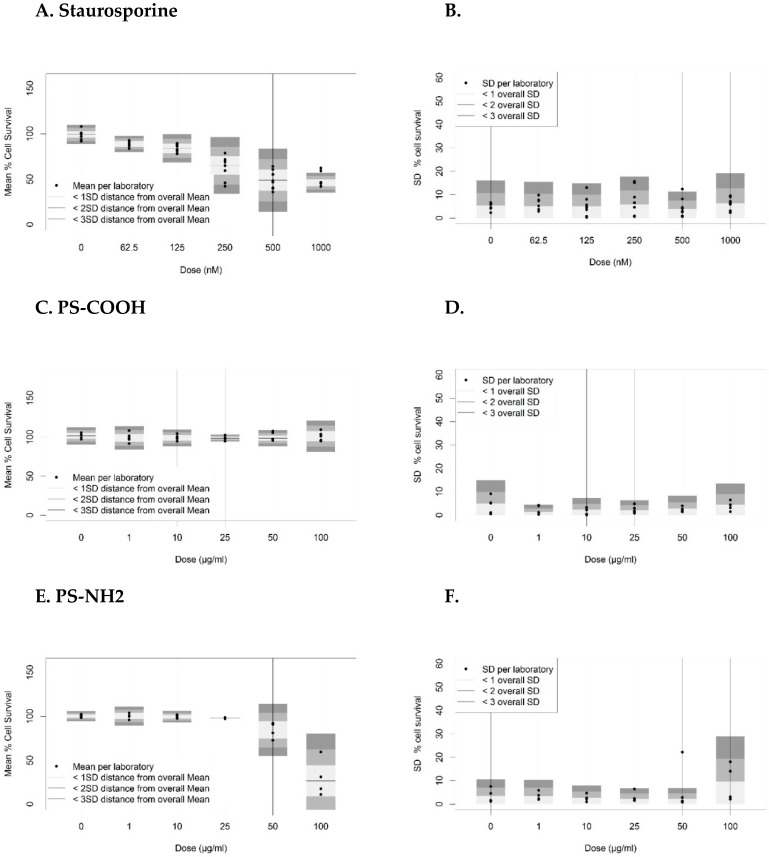
Intra- and inter-laboratory biases for percentage cell survival determined using the MTS assay based on valid runs (tier 2). Mean percentage cell survival per dose and per laboratory compared to the overall mean and SD (left panels), as well as SD of percentage cell survival per dose and per laboratory compared to the overall SD* (right panels) are shown for (**A**,**B**) staurosporine (N = 8), (**C**,**D**) carboxyl-modified polystyrene nanoparticles (PS-COOH) (N = 5) and (**E**,**F**) amine-modified polystyrene nanoparticles (PS-NH2) (N = 4). Horizontal bars (left panel) indicate overall mean values of percentage cell survival, while grey shaded areas indicate the distances from the overall mean corresponding to 1-, 2- or 3-fold the overall SD (left panels) or SD* (right panels).

The dose-response data of staurosporine and NP-induced cytotoxicity in A549 cells were evaluated using robust statistical methods. Examples of dose-response data from single laboratories are shown in Figure S3. Dose-dependent decrease in percentage cell survival was observed for staurosporine and PS-NH2, whereas PS-COOH did not affect cell viability as expected. Intra- and inter-laboratory biases were calculated, while including either all data from both valid and non-valid runs of all laboratories (Figure S4), or only the data from the laboratories with at least three valid runs (Figure 2).

Overall variability in the dose-response data, represented by the SD of the mean % cell survival of all laboratories and the SD*, which represents a pooled value of the within-laboratories’ SD, was observed to decrease when non-valid runs were discarded, indicating improved inter-laboratory reproducibility. At the same time, the number of laboratories with an intra-laboratory bias exceeding the overall mean plus 2-fold of the overall SD decreased (Figure 2 vs. Figure S4). These findings highlight the need to apply acceptance criteria as quality measures to the biological test performance to enhance reproducibility of results and hence standardization.

**Figure 3:**
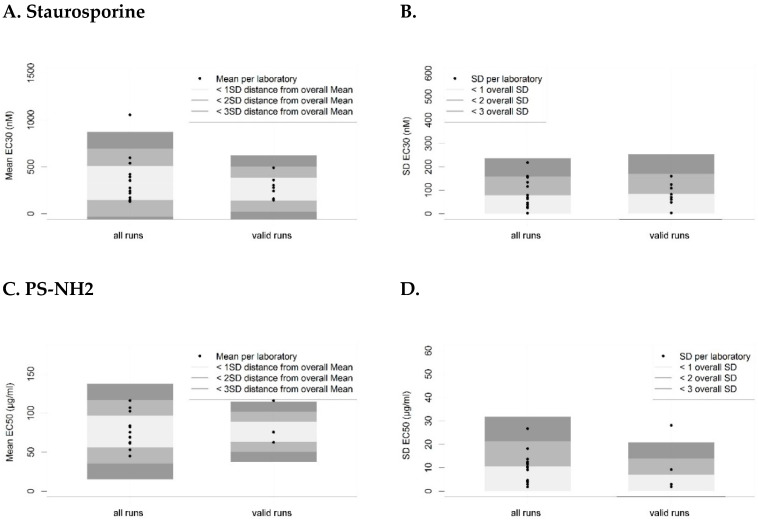
Intra- and inter-laboratory biases for effective concentration causing 30% inhibition of cell viability (EC30) and effective concentration causing 50% inhibition of cell viability (EC50) values determined using the MTS assay (tier 2). Mean values for EC30 and EC50 per laboratory compared to the overall mean and SD (left panels), as well as SD of EC30 and EC50 values per laboratory compared to the overall SD* (right panels) are shown for (**A**,**B**) staurosporine (N = 15 for all runs, N = 8 for valid runs) and (**C**,**D**) PS-NH2 (N = 15 for all runs, N = 4 for valid runs). Horizontal bars (left panel) indicate overall mean values of percentage cell survival, while grey shaded areas indicate the distances from the overall mean corresponding to 1-, 2- or 3-fold of the overall SD (left panels) or SD* (right panels).

To further assess the intra- and inter-laboratory bias of toxicity values obtained in the different laboratories, EC30 and EC50 values were derived from the fitted dose-response curves resulting from cell exposures to staurosporine and PS-NH2, respectively. Although an EC50 value can usually be derived more accurately than an EC30 value, approximately half of the participants were not able to observe 50% inhibition of cell viability at 1000 nM staurosporine. Therefore, the EC30 of staurosporine was reported and compared, since it corresponds to the threshold value for concluding on cytotoxicity according to ISO 10993-5:2009 [40]. When both non-valid and valid runs were taken into account, mean fitted EC30 of 329.7 ± 182.0 nM staurosporine and EC50 of 76.2 ± 20.4 µg/mL PS-NH2 were observed, with respective CVs of 55.2% for staurosporine and 26.8% for PS-NH2. These effective concentrations changed to an EC30 of 261.0 ± 121.1 nM staurosporine and EC50 of 76.2 ± 12.9 µg/mL PS-NH2 (Figure 3A,C) when only valid runs were considered, resulting in decreased CVs of 46.4% for staurosporine and 16.9% for PS-NH2, respectively. By excluding non-valid data the overall variability SD* of the EC50 values determined for PS-NH2 exposed cells was again decreased (10.6 for non-valid and valid data, vs. 6.9 for valid data; Figure 3D), indicating that intra-laboratory performances were also improved. In contrast, this improvement was not observed for EC30 determinations in tests with staurosporine (SD* of 78.7 vs. 84.6; Figure 3B), which, in general, showed lower reproducibility within and between laboratories. The latter is in agreement with the low stability of staurosporine in solution we observed during the study, and, therefore, other stable compounds, such as cadmium sulfate [34,37] are more suitable as a positive control.

Although variabilities were, in most cases, decreased when only valid results were included, these were still high (at the limit of what is acceptable) and warranted further investigation of possible causes and refinement of the benchmarking process. Thus, we examined in more detail if these first MTS experiments could highlight sensitive steps in the applied procedure, which introduced variability in the reported outcomes. This was done by collecting feedback on the interpretation of the SOP and performance of the MTS test by the participating laboratories using an online questionnaire. A list of critical steps that were reported in this survey is included in Table 2. These steps, including accurate pipetting volumes and seeding cell densities, were found to be quite similar to those already described in Rösslein et al. [36] and Elliott et al. [34].

Based on a thorough examination of the data and statistical analysis, and the collected feedback on critical phases in the test performance, we further optimized the SOP of the MTS cytotoxicity assay (available in the Supplementary Materials). One aspect concerned the possible contribution of cells and NPs present during assay read-out to variability in the absorbance values, frequently causing them to exceed a value of 2.0, which is outside the linear dynamic range that is relevant to applying the Lambert-Beer law, as previously suggested by Xia et al. [33]. Therefore, in the revised SOP, laboratories were asked to read out the assay plates in the presence and absence of cells. Other critical steps in the test performance which cannot simply be addressed in the SOP, such as the verification of instruments or pipetting techniques, constituted major challenges to be faced in this field of research, to ensure quality of results. This led us to organize a focused training to resolve these issues. Additionally, the majority of participating laboratories indicated that they were not familiar with the principles of an inter-laboratory comparison, as exemplified by the submission of a large number of non-valid data. Training was thus held by well documented instructions and a teleconference to introduce the relevant principles of GLP and proficiency testing, to transfer the optimized SOP and quality criteria, and to discuss in detail good practices for enhancing the test performance. Subsequently, the final tier of the ILC study was launched.

### 3.3. Laboratories’ Proficiency in Performing the In Vitro MTS Assay After Training (Tier 3)

In the third and final tier of the study, six laboratories were enrolled, of which five also had participated in the first phase (tier 1 and 2) with involvement of the same operator, and four had not been able to provide a full set of valid data in the second tier. The other laboratories dropped out mainly because of a shift or lack of resources at the time of the last tier. The participating laboratories were provided with fresh FBS and NPs, and used the optimized MTS assay SOP. All laboratories sent in data on three independent, valid runs performed on cells of different passage number from the same working cell bank vial. The dose-response data were evaluated for intra- and inter-laboratory biases, similar to the statistical analyses in tier 2 (Figures S5–S7). In Table 3, the mean ± SD values of EC30 and EC50 obtained by the six laboratories involved in the study before and after training are presented, together with the overall mean ± SD values of all laboratories.

When considering the entire data sets obtained in tier 2 (valid and non-valid runs) and tier 3 with read-out of the MTS assay in the presence of cells, the inter-laboratory variability assessed by means of CV (%) of all laboratories before (tier 2, N = 15) and after (tier 3, N = 6) training showed improvement due to training (respectively 55.2% compared to 49.6% for staurosporine, and 26.8% compared to 23.0% for PS-NH2). Furthermore, the reproducibility within the laboratories was found to be increased after training, which is obvious from the decrease in CV per laboratory (example of dose-response curves in Figure S8), as well as the decrease of overall SD* in the case of staurosporine (Table 3). Finally, reading out the MTS assay in the absence of cells resulted in a mean fitted EC30 of 264.3 ± 140.0 nM staurosporine and EC50 of 80.7 ± 22.8 µg/mL PS-NH2. Although this additional step did not further decrease the inter-laboratory variability (CV of all laboratories of 53.0% for staurosporine, and 28.2% for PS-NH2), it did further improve intra-laboratory performances for most laboratories as compared to cells present in the wells, evident again by decreased CV of individual laboratories and SD* (Figure S6 vs. Figure S5, Figure S7, and Table 3). Taking into account also the fact that in the second tier the majority of laboratories sent in non-valid run data (Table 1), because they were ignorant of good practice and ILC principles, these comparative analyses show that training improved the laboratories’ proficiencies and ILC results.

## 4. Discussion

Nanotoxicological data reported in the literature have shown low reproducibility within and between different studies, and, consequently, conflicting conclusions. In the current ILC study, we aimed to tackle this problem by introducing SOPs for cytotoxicity testing via the MTS assay, using previously well characterized control NMs. In particular, we have evaluated the impact of training in these SOPs and in good laboratory practices on the proficiency of the participating laboratories.

The ILC study was composed of three tiers, including cell culturing and cell growth rate determination (tier 1), MTS assay for cytotoxicity measurement of NMs to evaluate the laboratories’ inherent performance (tier 2) and, finally, a repetition of the MTS assay after training of the laboratories (tier 3). The process applied in this study has highlighted the degree of complexity that is related to good practice in nanosafety testing, where multiple consecutive steps of the workflow should all be tightly aligned, including material storage, NP dispersion, cell culture, cell seeding and exposure, and test performance. This has also been concluded in a round robin study performed by different partner laboratories of the German Priority Programme SPP1313, who observed that small variations in NP preparation, cell handling and the type of culture slide influenced NP stability and the outcomes of cell assays [57]. In another ILC study on the MTS cytotoxicity assay [34], system control measurements revealed similar steps in the protocol that are critical to ensure overall robustness and reproducibility of the assay results within and between laboratories. These factors have also been taken into account in ISO 19007:2018(E) [37].

In contrast to previous interlaboratory studies in the nanosafety research field, we here demonstrated that the availability of an optimized SOP in combination with training and the active implementation of good practice in test performance enhanced the quality of intra-laboratory test results, and improved inter-laboratory variability. The ILC study has also highlighted that principles of GLP, including the use of verified instruments and registration of each step in the execution, may provide guidance to enhance quality of results from in vitro toxicity assays. The developed SOPs for cell culture, cell growth rate determination and the MTS assay for the cytotoxicity assessment of NMs, were compiled to cover the different categories of test facility activities, including equipment, media and reagents, test and control items, the consecutive steps of the experimental protocol in a chronological order and data analysis. Furthermore, the SOPs included acceptance criteria to monitor test performance, forms to record the laboratory performance and observations, and calculation templates for the reporting of the test data. Each of these categories contained sufficient and explicit detail to ensure proper execution. Based on feedback collected from the participating laboratories through an online survey, however, we found that despite the availability of SOPs, deviations in the execution were frequently reported, for example, in the verification of laboratory instruments or the addition of antibiotics to the cell culture medium. Additionally, basic laboratory practices, such as pipetting techniques, which are not normally made explicit in SOPs were identified as a potential source of variability, in addition to influences from differences in technical infrastructure. This overall complexity accounts for the conflicting results on NPs’ toxicity in the reported literature, and calls for increasing awareness and proper training of nanosafety professionals. Based on the feedback received after the second tier, the MTS assay SOP was revised to account for the identified hidden sources of variability, and additional training was provided to ensure all participating laboratories were familiar with the procedures and carefully followed all steps as detailed in the SOP. The results of the second ILC on the MTS assay showed that this process makes it possible to obtain higher reproducibility across independent laboratories from academia, research institutions and industry. The NIEHS Nano GO Consortium [33] has previously investigated NMs in several bioassays, including the MTS assay. They also found substantial variations in the results between different laboratories, and further stated that “frequent communication was very helpful for achieving reproducible results within and among the laboratories”. However, no further details regarding the improvement of reproducibility were given. As similar issues related to the quality in test performance discussed in our study are also present among different operators within the same laboratory, it may be valuable to implement a similar process using shared materials as described here within laboratories, to determine and align the reproducibility in performance by multiple operators. A representative selection of positive and negative control materials, as well as SOPs with quality acceptance criteria and statistical data analysis, will make it possible to judge whether the data generated by multiple operators, even within a single laboratory, are reliable or not.

As a secondary outcome of this ILC study, we confirmed the suitability of PS-COOH and PS-NH2 as negative and positive control nanomaterials, respectively, to validate the test performance of the MTS assay. The PS-COOH NPs are complementary to other suggested negative nano-sized control materials for in vitro cell viability assays, such as barium sulfate [44] and carboxylated nanodiamonds [45]. Positively charged PS-NH2 NPs have already been included as validated positive control nanomaterials in the ISO 19007:2018(E) standard [37]. Based on the dose-response data obtained for the PS-NH2 in our large ILC study comprising a representative sample of research laboratories, an EC50 value of 80.7 ± 22.8 µg/mL was obtained which is exactly within the commonly accepted biological variability window of CV <30%. This EC50 for PS-NH2 was higher than the consensus EC50 value of 52.6 µg/mL (95% confidence intervals 44.1 to 62.6 µg/mL) for the same NPs in the A549 cell line reported by Elliott et al. [34], which can be attributed to differences in cell stocks, serum sources, cell seeding density and exposure times (24 vs. 48 h, respectively). Nevertheless, variability in the EC50 values in both studies was in a similar range and well below 30%, confirming the suitability of 50-nm PS-NH2 as positive control NPs in the MTS cytotoxicity assay. However, it should be mentioned that, in both studies, PS-NH2 from Bangs Laboratories Inc. have been used, which were not available anymore from the supplier at the time of this report. Therefore, to enable standardization in nanosafety testing, stable and effective reference NPs from a secured source are urgently needed.

We also stress that for this study, in order to focus—as a first step—on the sources of variability related to cell toxicity testing, we selected model PS NPs behaving well in terms of stability and dispersion, thus, many of the reproducibility issues highlighted in our study are not nano-specific. Reproducibility in in vitro nanotoxicity testing goes far beyond this simplistic view, as real nanoparticles, such as, for instance, metal oxides, introduce many more challenges related to their intrinsic (medium independent) and extrinsic (medium dependent) physical and chemical properties, which affect their dispersion and stability in cell culture medium, and—as a consequence of this—their fate and transport into cells, and, thus, the dose delivered to cells as a function of exposure time [58,59]. For example, surface affinity, which is dependent on particle and medium parameters, may cause the agglomeration or aggregation of nanomaterials, whereas particle size and density can affect the diffusion and sedimentation of NPs, thus affecting their in vitro exposure. Furthermore, especially for partially soluble NPs, such as metal and metal oxides, the rate of release of ions is depending on system parameters (e.g., pH, ligands present, flow conditions, etc.) and can greatly influence their in vitro toxicity potential. To allow for the correct interpretation of in vitro assay data using NPs closer to real applications, as opposed to model NPs with optimal dispersibility, standard protocols for the dispersion of nanomaterials in complex media, dispersion characterization and dosimetry, as described by Deloid et al. [59] and in ISO/TR 16196:2016 [60], should be adopted to ensure meaningful and reproducible quantification of in vitro delivered dose. The standardization of characterization methods to monitor the physicochemical properties before and after NPs’ dispersion is also work in progress, e.g., by the OECD Series on the Safety of Manufactured Nanomaterials [58] and ISO/TC229 (Nanotechnologies), and has been subject of several ILC studies. Remarkably, in ILC studies in which model nanoparticles as polystyrene, gold or silica nanospheres have been used, for example those looking at size distribution measurements using nanoparticle tracking analysis [61], dynamic light scattering and centrifugal particle sedimentation [49], similar issues in reproducibility compared to our study have been reported, even for simple dispersions in buffer. They concluded that SOPs are indispensable for obtaining reliable and comparable NP size data, and should be tailored to the specific test system, sufficiently detailed and verified by a larger number of laboratories, to enable reproducible performance [49,61].

Other NP types than polystyrene have already been applied by others in the MTS assay, such as titanium dioxide, zinc oxide and multi-walled carbon nanotubes [33]. In addition to selecting the best protocol for NPs’ dispersion and verification of the dispersion stability in complete CCM, as indicated above, these will require a case-by-case consideration of interference of the NPs with the optical density measurements. Although, initially, a number of laboratories observed optical interference of PS-NH2 with the MTS test read-out in our study (tier 2), we showed that, after good practice training and diligent application of the optimized SOP, this bias was resolved. This may be attributed to detailed instructions on NP dispersion in serum-containing medium and the use of the same serum batch by all laboratories. In addition, we introduced in the SOP a transfer of MTS medium to a new plate before optical read-out to avoid high absorbance contribution of the cells and NPs remaining in the wells. In this respect, our SOP deviates from the ILC study of Elliott et al. [34] and the ISO 19007:2018(E) standard [37], in which the average background absorbance level of the NP doses in culture medium are subtracted from each absorbance value of NP-exposed cells. In contrast, we recommend using an alternative cytotoxicity assay, based on a different optical read-out principle, in case NP interference is observed, as the correct assessment of the issue and adjustment is hampered by multiple factors, such as NP agglomeration, NP adherence to the cell surface or assay plate, cell-dependent NP uptake kinetics, etc.

Finally, it remains to be investigated whether the MTS assay SOP developed and tested by multiple laboratories in our study can be transferred to other cell types. Based on our experience, the SOP works optimally for adherent cell types growing in monolayers. The SOP can be adapted for use with suspension cells by introducing centrifugation steps to pellet the cells at the bottom of the wells before medium changes.

In conclusion, the experience reported in this study overall clearly indicates that the approach followed in this study, including inter-laboratory comparison studies using shared materials and detailed SOPs, together with training of the participants, can be used to optimize and generate robust SOPs, and to obtain reproducible data on NP cytotoxicity within and across independent laboratories.

## Figures and Tables

**Table 1:** Compliance of laboratories with the acceptance criteria of the 3-(4,5-dimethylthiazol-2-yl)-5-(3-carboxymethoxyphenyl)-2-(4-sulfophenyl)-2H-tetrazolium (MTS) cytotoxicity assay. The number of compliant laboratories compared to the total number of laboratories is indicated per test item.

Acceptance Criteria	Staurosporine	PS-COOH	PS-NH2
- Average absorbance values of NP blank deviating <15% from medium blank (no NP interference)	n/a ^1^	14/15	8/15
- Blank replicate values, CV ^2^ <30%	15/15	15/15	15/15
- Blank-corrected absorbance values >0.1	15/15	15/15	14/15
- Blank-corrected absorbance values, CV <30%	12/15	14/15	6/15
- Average absorbance values at zero dose deviating <15% from non-treated cells	8/15	6/15	7/15
- % cell survival <70% for at least one concentration of positive chemical control (staurosporine)	13/15	14/15	13/15
**≥ three valid and independent runs**	**8/15**	**5/15**	**4/15**

^1^ n/a, not applicable; ^2^ CV, coefficient of variation.

**Table 2:** Critical steps in the standard operating procedure (SOP) for the MTS cytotoxicity assay. Following the analysis of the first data obtained for cells treated with staurosporine, PS-NH2 and PS-COOH, and the collection of responses of the participating laboratories to an online questionnaire on SOP interpretation and test performance, sensitive steps in the SOP that could generate and explain the observed variability of outcomes have been individuated.

Protocol Step	Critical Phase
All steps	- Verification of pipets and instruments - Use of single vs. multi-channel pipets - Pipetting technique - Adherence to timings stated in the SOP
Preparation and storage of staurosporine stock	- Dissolution of lyophilized product
Preparation of staurosporine working solution	- Low pipetting volume
Preparation of NP dilutions in cell culture medium	- Low pipetting volume - Dispersion protocol
Preparation of dosing plate	- Different pipetting volumes
Plating cells	- Use of antibiotics-free cell culture medium - Cell counting method - Homogeneous suspension of cells - Edge effects
Exposure to test item	- Removal of medium from cultures - Homogeneous suspension of test items - Application of test solutions onto cultures
MTS assay	- Removal of medium from cultures - Air bubbles - Precipitate of MTS reagent - Transfer of MTS reagent for read-out - Spectrophotometer specifications

**Table 3:** Comparison of intra- and inter-laboratory biases for EC30 and EC50 values before (tier 2) and after (tier 3) training in quality aspects of MTS assay performance. Individual data from the six laboratories that were trained, as well as the overall data of all laboratories (N; in bold and between brackets) are included. Mean and SD (and SD*, in bold and between brackets) values of calculated EC30 (nM staurosporine) and EC50 (µg/mL PS-NH2), coefficient of variation (CV) (%) and number of runs (n) are given. For tier 2, data from all (valid and non-valid) runs and valid runs only are indicated. Tier 3 data are presented in the presence and absence of cells. ‘-’ indicates that no data were available, laboratory 6 participated only in tier 3.

		EC30 Staurosporine (nM)				EC50 PS-NH2 (µg/mL)			
	Laboratory	Mean	SD	CV (%)	Runs (n)	Mean	SD	CV (%)	Runs (n)
**Before training (tier 2)** **All runs**	1	539.9	63.7	11.8	2	83.7	12.1	14.4	8
	2	421.3	218.6	51.9	16	82.2	26.8	32.5	9
	3	275.0	79.4	28.9	7	75.6	9.2	12.2	4
	4	171.4	28.9	16.9	10	62.7	10.2	16.2	5
	5	129.4	2.4	1.8	4	69.4	10.5	15.1	2
	6	-	-	-	-	-	-	-	-
	**All labs**	**329.7 (N = 15)**	182.0 (SD* = 78.7)	**55.2**	**-**	**76.2 (N = 15)**	20.4 (SD* = 10.6)	**26.8**	**-**
**Before training (tier 2)** **Valid runs**	1	-	-	-	-	75.9	28.1	37.0	3
	2	303.4	159.9	52.7	6	-	-	-	-
	3	278.8	108.2	38.8	5	75.6	9.2	12.2	4
	4	160.1	81.8	51.1	5	-	-	-	-
	5	-	-	-	-	-	-	-	-
	6	-	-	-	-	-	-	-	-
	**All labs**	**261.0 (N = 8)**	121.1 (SD* = 84.6)	**46.4**	**-**	**76.2 (N = 4)**	12.9 (SD* = 6.9)	**16.9**	**-**
**After training (tier 3)** **Cells present**	1	78.1	18.3	23.4	6	59.5	7.6	12.8	3
	2	237.3	73.4	30.9	6	102.8	20.0	19.4	3
	3	156.2	23.6	15.1	6	71.3	3.9	5.4	3
	4	255.0	92.9	36.4	3	87.5	21.1	24.2	3
	5	290.4	40.4	13.9	6	87.5	9.5	10.9	3
	6	596.1	194.5	32.6	6	103.2	21.6	21.0	3
	**All labs**	**238.5 (N = 6)**	118.3 (SD* = 59.4)	**49.6**	**-**	**85.3 (N = 6)**	19.7 (SD* = 16.5)	**23.0**	**-**
**After training (tier 3)** **Cells absent**	1	82.9	11.1	13.4	6	56.9	4.7	8.2	3
	2	267.2	71.4	26.7	6	104.5	0.2	0.2	3
	3	162.0	21.5	13.3	6	70.5	4.8	6.9	3
	4	320.2	90.9	28.4	3	64.1	9.5	14.8	3
	5	284.6	29.4	10.3	6	85.3	8.9	10.4	3
	6	581.8	143.9	24.7	6	102.6	30.7	29.9	3
	**All labs**	**264.3 (N = 6)**	140.0 (SD* = 53.1)	**53.0**	**-**	**80.7 (N = 6)**	22.8 (SD* = 7.7)	**28.2**	**-**

## Supplementary Materials

The following are available online at https://www.mdpi.com/2079-4991/10/8/1430/s1, Figure S1: Dosing Plate Layout for the MTS assay, Figure S2: Growth curve of the A549 cell line, Figure S3: Dose-response curves of cytotoxicity assessment using the MTS assay (Tier 2) from two individual laboratories, Figure S4: Intra- and inter-laboratory biases for percentage cell survival determined using the MTS assay based on all (valid and non-valid) runs of all laboratories (tier 2), Figure S5: Intra- and inter-laboratory biases for percentage cell survival determined using the MTS assay based on valid runs of laboratories after training in quality aspects of assay performance (tier 3), Figure S6: Intra- and inter-laboratory biases for percentage cell survival determined using the MTS assay based on valid runs of laboratories after training in quality aspects of assay performance (tier 3), Figure S7: Intra- and inter-laboratory biases for EC30 and EC50 values determined using the MTS assay based on valid runs of laboratories after training in quality aspects of assay performance (tier 3), Figure S8: Comparison of intra-laboratory bias in cytotoxicity assessment using the MTS assay before (tier 2) and after training in quality aspects of assay performance (tier 3), Standard Operating Procedure for A549 cell culturing, Standard Operating Procedure for assessment of A549 cell growth rate and viability, Calculation template for assessment of A549 cell growth rate and viability, Standard Operating Procedure for the MTS cytotoxicity assay, Calculation template for the MTS cytotoxicity assay.

## Abbreviations

The following abbreviations are used in the manuscript:

CCM	Cell culture medium
CV	Coefficient of variation
DMSO	Dimethyl sulfoxide
EC30	Effective concentration causing 30% inhibition of cell viability
EC50	Effective concentration causing 50% inhibition of cell viability
FBS	Fetal bovine serum
GLP	Good laboratory practice
ILC	Inter-laboratory comparison
ISO	International Organization for Standardization
MTS	3-(4,5-dimethylthiazol-2-yl)-5-(3-carboxymethoxyphenyl)-2-(4-sulfophenyl)-2H-tetrazolium
MTT	3-(4,5-dimethylthiazol-2-yl)-2,5-diphenyltetrazolium bromide
NM	Nanomaterial
NP	Nanoparticle
OECD	Organization for Economic Cooperation and Development
PS-COOH	Carboxyl-modified polystyrene nanoparticles
PS-NH2	Amine-modified polystyrene nanoparticles
SD	Standard deviation
SOP	Standard operating procedure
