# Supplementary material for: Improving Quality in Nanoparticle-Induced Cytotoxicity Testing by a Tiered Inter-Laboratory Comparison Study
Source: Nanomaterials (Basel). 2020 Jul 22;10(8):1430. doi: 10.3390/nano10081430 (PMC7466672; doi:10.3390/nano10081430)

nanomaterials

Supplementary Materials

Improving Quality in Nanoparticle-Induced Cytotoxicity Testing by a Tiered Inter-Laboratory Comparison Study

Inge Nelissen*, Andrea Haase, Sergio Anguissola, Louise Rocks, An Jacobs, Hanny Willems, Christian Riebeling, Lisa Wollenberg, Jean-Pascal Piret, Olivier Toussaint, Bénédicte Trouiller, Ghislaine Lacroix, Arno C. Gutleb, Servane Contal, Silvia Diabaté, Carsten Weiss, Tamara Lozano-Fernández, África González-Fernández, Maria Dusinska, Anna Huk, Vicki Stone, Nilesh Kanase, Marek Nocuń, Maciej Stępnik, Stefania Meschini, Maria Grazia Ammendolia, Nastassja Lewinski, Michael Riediker, Marco Venturini, Federico Benetti, Jan Topinka, Tana Brzicova, Silvia Milani, Joachim Rädler, Anna Salvati, Kenneth A. Dawson

*Correspondence should be addressed to:
Inge Nelissen
Flemish Institute for Technological Research (VITO)
Health Department
Boeretang 200
BE-2400 Mol
Belgium
Email: inge.nelissen@vito.be
Phone: +32 14 33 55 11

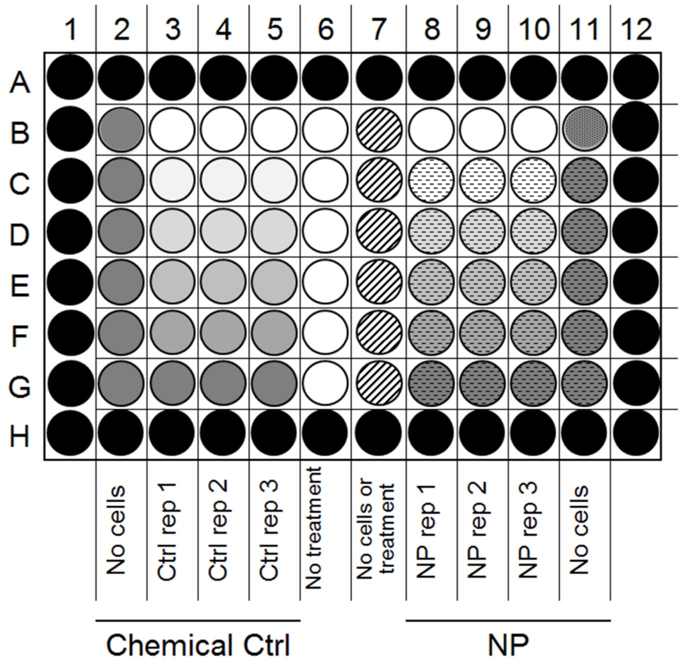

Staurosporine (nM)	NP (µg/ml)
0	0
62.5	1
125	10
250	25
500	50
1000	100

Figure S1. Dosing Plate Layout for the MTS assay. The white and hatched wells contain complete CCM +1% milliQ water, the black wells contain medium to avoid electrostatic effects. Columns 3-5 and columns 8-10 contain chemical and NP concentration series, whereas columns 2 and 11 contain a single concentration of the chemical blank (1000 nM staurosporine) and NP blank (100 µg/ml NPs), respectively (adapted with permission from original figure of Rösslein et al. 2015; http://pubs.acs.org/doi/full/10.1021/tx500327y).

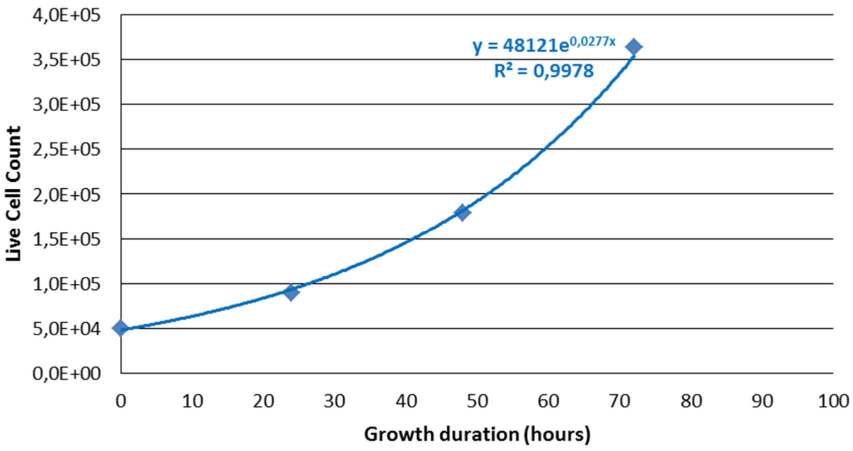

Figure S2. Growth curve of the A549 cell line. Cells were seeded in 24-well plates, cultured for 24, 48 and 72 hours, and counted. Exponential curve fit of live cell counts plotted against the growth time is shown. The prefactor of the exponential power from the equation indicating the relative growth rate (doublings per hour) was used to calculate the cell doubling time.

.

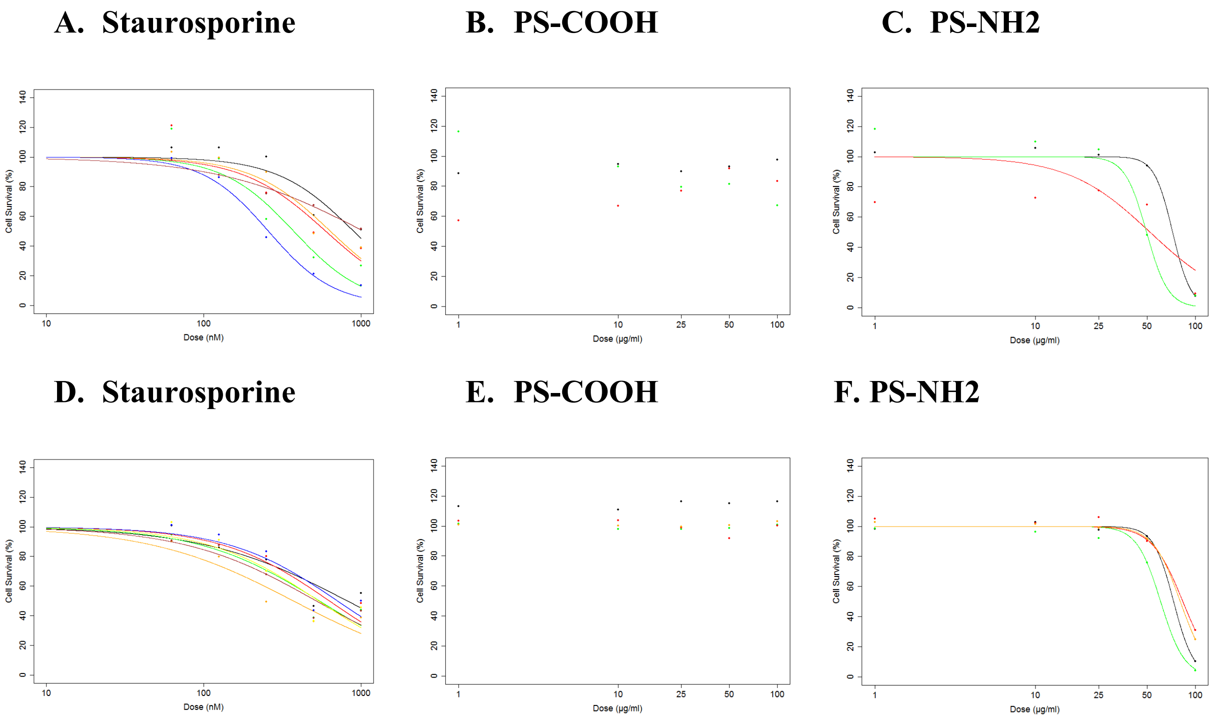

Figure S3. Dose-response curves of cytotoxicity assessment using the MTS assay (Tier 2) from two individual laboratories. Fitted sigmoidal curves of percentage cell survival following exposure to (**A,D**) staurosporine (nM), (**B,E**) PS-COOH (µg/ml) and (**C,F**) PS-NH2 (µg/ml) are shown. Each curve represents the data of an independent run. The upper panels (A-C) show example runs of one laboratory which was not proficient in testing; the lower panels (D-F) show valid runs of a different laboratory which proved to be proficient.

A. Staurosporine

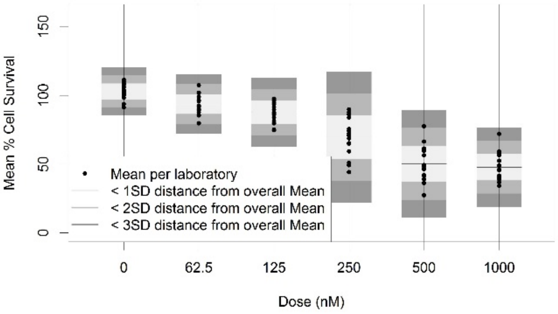

B.

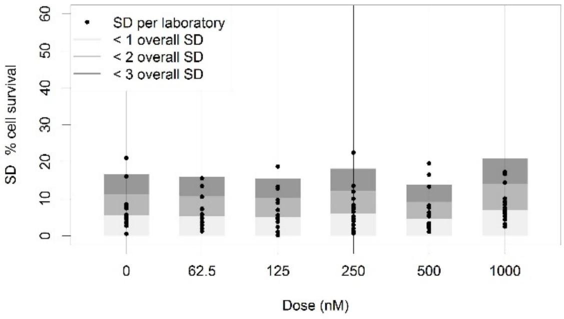

C. PS-COOH

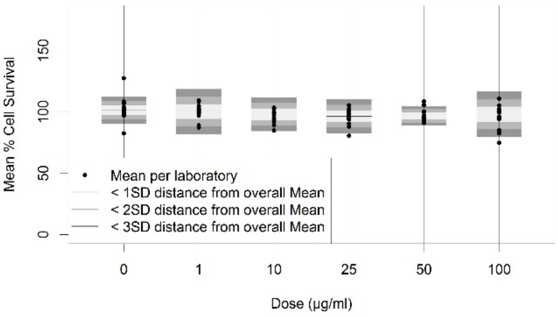

D.

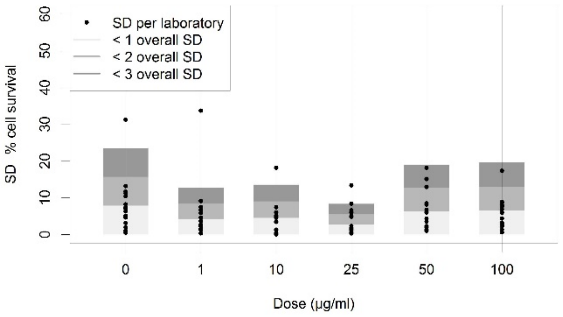

E. PS-NH2

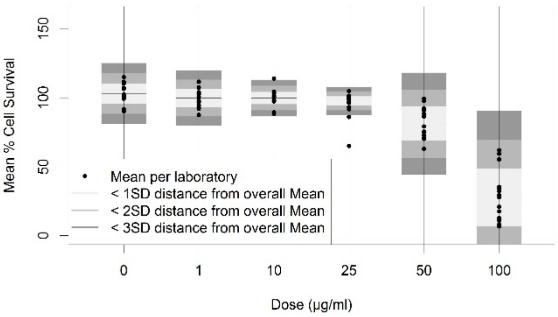

F.

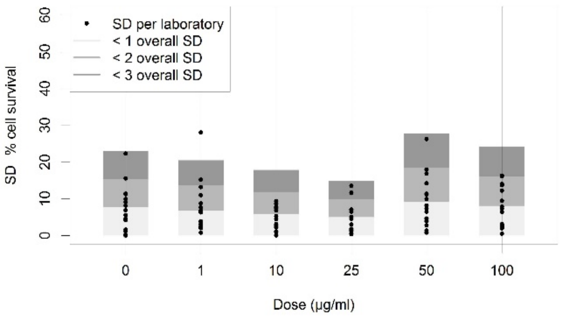

Figure S4. Intra- and inter-laboratory biases for percentage cell survival determined using the MTS assay based on all (valid and non-valid) runs of all laboratories (tier 2). Mean percentage cell survival per dose and per laboratory compared to the overall mean and SD (left panels), as well as SD of percentage cell survival per dose and per laboratory compared to the overall SD* (right panels) are shown for (**A,B**) staurosporine (N=15), (**C,D**) PS-COOH (N=15) and (**E,F**) PS-NH2 (N=15). Horizontal bars (left panel) indicate overall mean values of percentage cell survival, while grey shaded areas indicate the distances from the overall mean corresponding to 1-, 2- or 3-fold the overall SD (left panels) or SD* (right panels).

.

A. Staurosporine

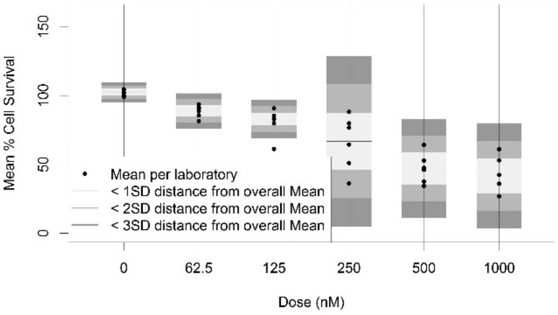

B.

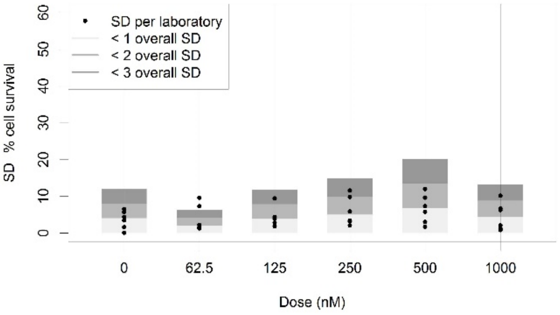

C. PS-COOH

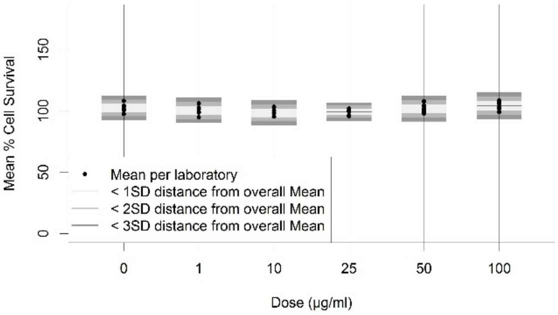

D.

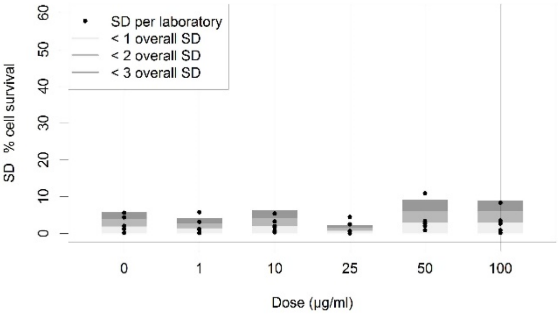

E. PS-NH2

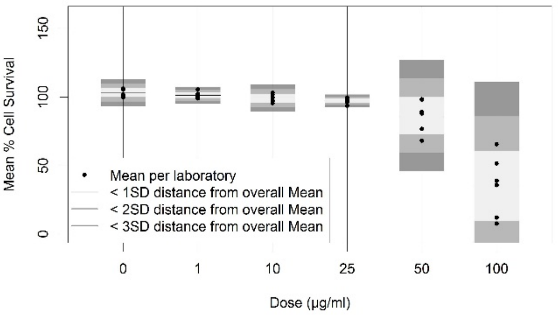

F.

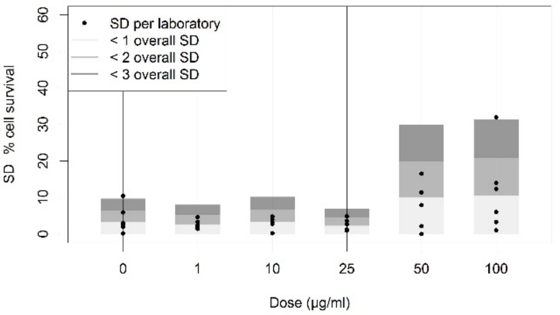

Figure S5. Intra- and inter-laboratory biases for percentage cell survival determined using the MTS assay based on valid runs of laboratories after training in quality aspects of assay performance (tier 3). Results based on absorbance measurements in the presence of cells are shown. Mean percentage cell survival per dose and per laboratory compared to the overall mean and SD (left panels), as well as SD of percentage cell survival per dose and per laboratory compared to the overall SD* (right panels) are shown for (**A,B**) staurosporine (N=6), (**C,D**) PS-COOH (N=6) and (**E,F**) PS-NH2 (N=6). Horizontal bars (left panel) indicate overall mean values of percentage cell survival, while grey shaded areas indicate the distances from the overall mean corresponding to 1-, 2- or 3-fold the overall SD (left panels) or SD* (right panels).

.

A. Staurosporine

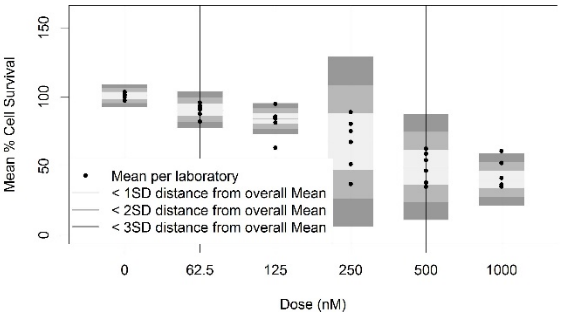

B.

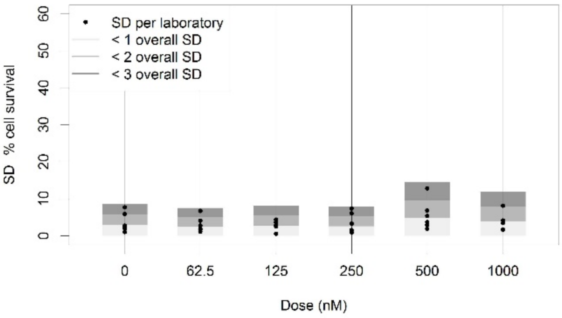

C. PS-COOH

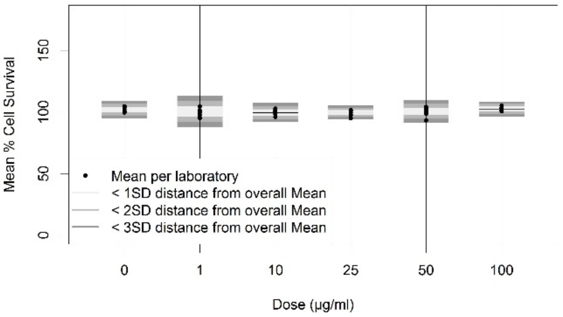

D.

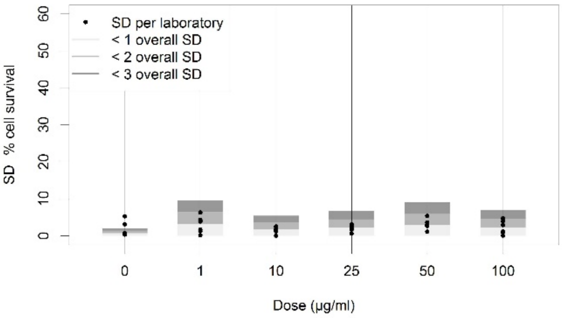

E. PS-NH2

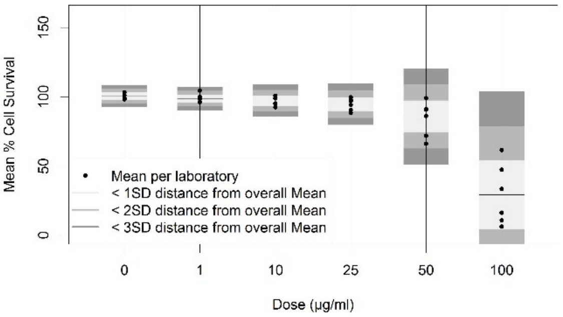

F.

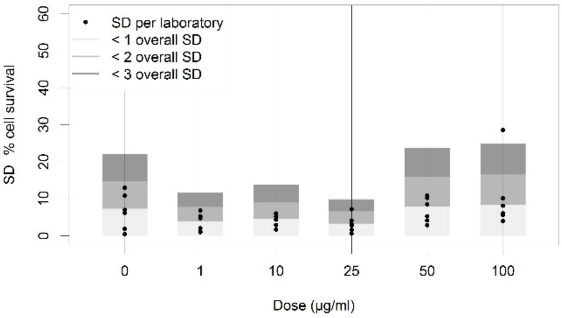

Figure S6. Intra- and inter-laboratory biases for percentage cell survival determined using the MTS assay based on valid runs of laboratories after training in quality aspects of assay performance (tier 3). Results based on absorbance measurements in the absence of cells are shown. Mean percentage cell survival per dose and per laboratory compared to the overall mean and SD (left panels), as well as SD of percentage cell survival per dose and per laboratory compared to the overall SD* (right panels) are shown for (**A,B**) staurosporine (N=6), (**C,D**) PS-COOH (N=6) and (**E,F**) PS-NH2 (N=6). Horizontal bars (left panel) indicate overall mean values of percentage cell survival, while grey shaded areas indicate the distances from the overall mean corresponding to 1-, 2- or 3-fold the overall SD (left panels) or SD* (right panels).

.

A. Staurosporine

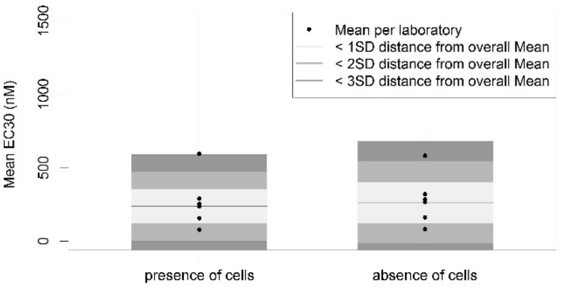

B.

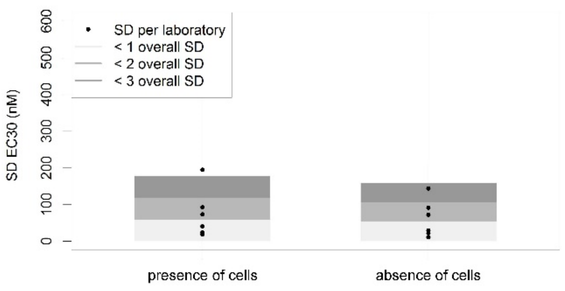

C. PS-NH2

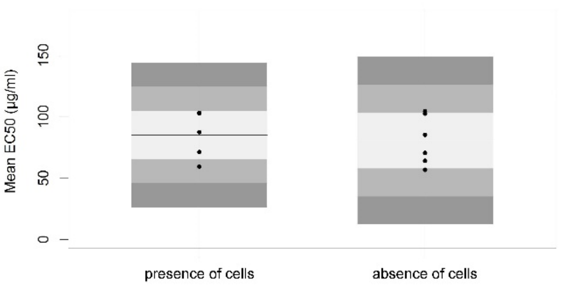

D.

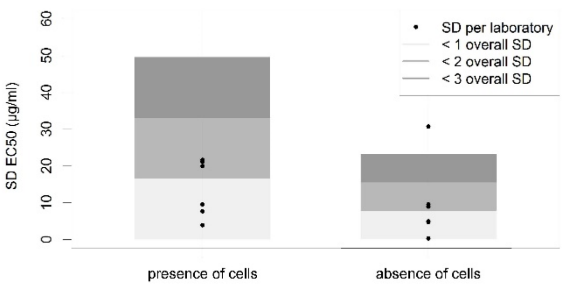

Figure S7. Intra- and inter-laboratory biases for EC30 and EC50 values determined using the MTS assay based on valid runs of laboratories after training in quality aspects of assay performance (tier 3). Results based on absorbance measurements in the presence and absence of cells are shown. Mean values for EC30 and EC50 per laboratory compared to the overall mean and SD (left panels), as well as SD of EC30 and EC50 values per laboratory compared to the overall SD* (right panels) are shown for (**A,B**) staurosporine (N=6) and (**C,D**) PS-NH2 (N=6). Horizontal bars (left panel) indicate overall mean values of percentage cell survival, while grey shaded areas indicate the distances from the overall mean corresponding to 1-, 2- or 3-fold the overall SD (left panels) or SD* (right panels)

Tier 2

A. Staurosporine

B. PS-COOH

C. PS-NH2

EC30 = 278,8 ± 108,2 nM (38,8%)

EC50 = 75,6 ± 9,2 µg/ml (12,2%)

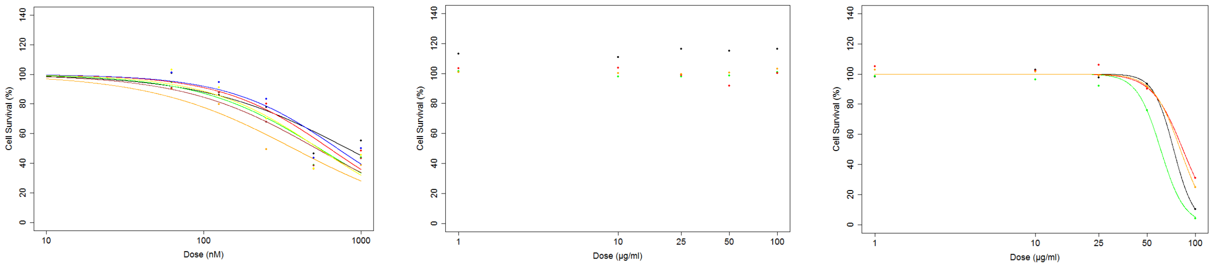

Tier 3 – Read-out in presence of cells

D. Staurosporine

E. PS-COOH

F. PS-NH2

EC30 = 156,2 ± 23,6 nM (15,1%)

EC50 = 71,3 ± 3,9 µg/ml (5,4%)

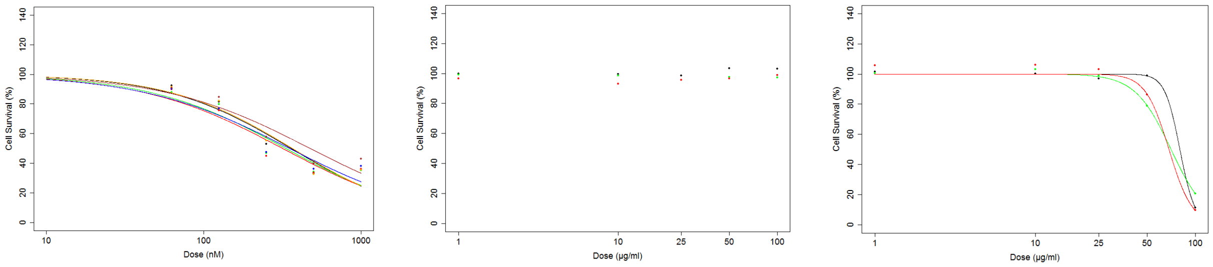

Tier 3 – Read-out in absence of cells

G. Staurosporine

H. PS-COOH

I. PS-NH2

EC30 = 162,0 ± 21,5 (13,3%)

EC50 = 70,5 ± 4,8 µg/ml (6,9%)

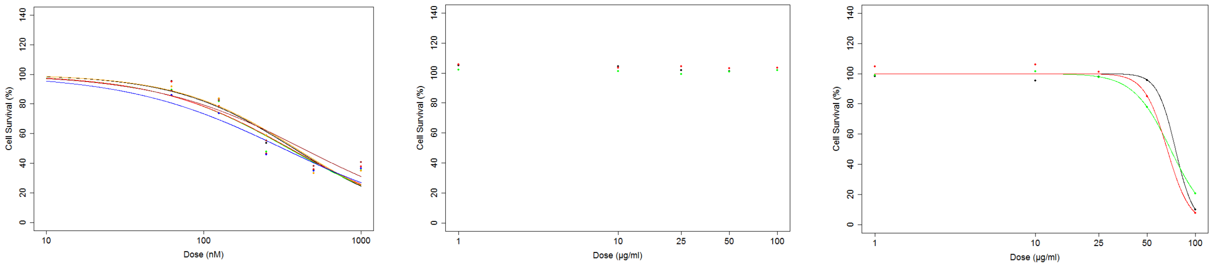

Figure S8. Comparison of intra-laboratory bias in cytotoxicity assessment using the MTS assay before (tier 2, upper panels) and after training in quality aspects of assay performance (tier 3 middle and lower panels; D-F, read-out in presence of cells; G-I, read-out in absence of cells). Dose-response curves from a single laboratory proficient in testing are shown. Fitted sigmoidal curves of percentage cell survival following exposure to (**A,D,G**) staurosporine (nM), (**B,E,H**) PS-COOH (µg/ml) and (**C,F,I**) PS-NH2 (µg/ml) are shown. Each curve represents the data of an independent run. Mean EC30 (N=6) and EC50 (N=3) ± SD, as well as calculated CV (%, between brackets) are indicated above the graphs for staurosporine and PS-NH2, respectively.

Standard Operating Procedure for A549 cell culturing

1. Introduction

The A549 cell line is a human cancerous cell line originated from lung carcinomatous tissue from a 58-year-old Caucasian male. The cells have a epithelial morphology and are adherent.

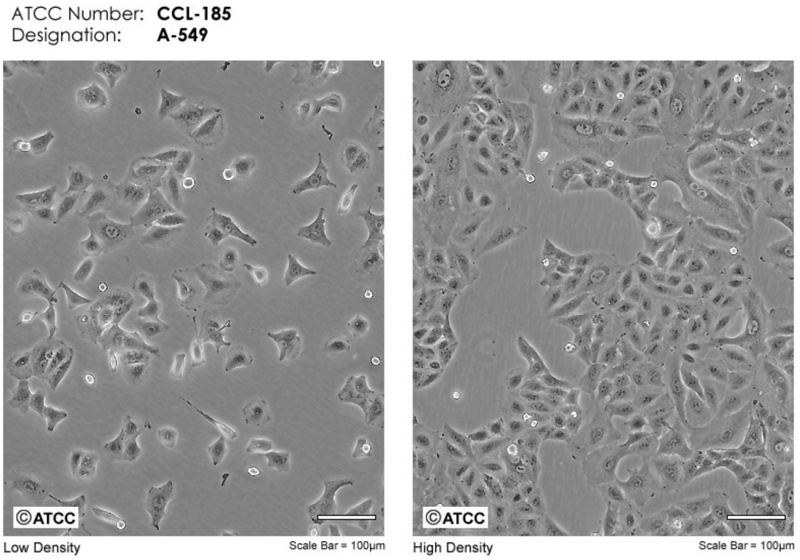

This SOP describes the procedures for thawing, freezing and subculturing the A549 cells.

2. Safety measures

- In-house procedures for safe handling of liquid nitrogen should be followed.
- All work should be performed in a laminar flow biosafety cabinet to ensure sterile handling of cell cultures.
- The cell line should be handled as a biosafety level 1 organism. This means e.g. that cells should be manipulated under laminar flow biosafety cabinet and the technician should wear nitrile gloves.
- All disposable materials, the remainder of the stock solutions, the content of the waste container connected to the aspirator and the remainder of the cells must be removed as toxic biologically hazardous waste.

3. REAGENTS AND EQUIPMENT

3.1. REAGENTS

- A549 cell stock (frozen vial)
- Modified Eagle Medium (MEM) with glutamax (Invitrogen, Cat. # 41090093)
- Fetal Bovine Serum (FBS), non-heat inactivated
- Penicillin/streptomycin (Invitrogen Cat.# 15070063)
- 0.05% Trypsin-EDTA (Invitrogen Cat.# 25300054)
- Phosphate Buffer Saline (PBS) sterile, without Ca^{2+}/Mg^{2+}
- DMSO, cell biology grade
- Isopropanol (for freezing container)

3.2. EQUIPMENT

- 50 ml and 15 ml sterile conical polystyrene tubes
- Water bath
- Liquid nitrogen tank
- Freezing container
- Sterile cryotubes (2ml)
- Micropipettors* + sterile tips
- Biohazard
- CO_2 incubator[1] at 37±2 °C, 5±0.5 % CO_2, 95 % relative humidity
- Centrifuge
- Aspirator + waste container
- Light microscope
- Refrigerator(4 °C) and Freezer (-20 °C)
- T75 (75 cm^2) and T25 (25 cm^2) tissue culture flasks, polystyrene, vented
- Cell counter[2] (e.g. Countess Cell Counter, Invitrogen)
- Pipette aid
- 100 ml sterile glass bottles
- Single wrapped sterile 5, 10 and 25 ml serological pipettes

[1]Verify that the incubator has been recently calibrated for temperature, humidity and CO_2 concentration. [2]In case an automated cell counting system is used, make sure its performance has been verified according to the manufacturer's recommendations before measurement.

4. MEDIA

4.1. Complete cell culture medium (CCM)

Composition:
MEM with glutamax
(100 U/ml Penicillin)
(100 μg/ml streptomycin)
10% FBS
Note: Complete CCM needs to be stored at 4°C.

Note: The use of antibiotics-free CCM is preferred for cell culture. Only during exposure experiments antibiotics can be used.

4.2. Freezing medium

Composition:
20% DMSO
80% FBS
Note: Freezing medium is freshly prepared before each use.

4.3. FBS aliquots

Upon starting a new bottle of FBS stock, aliquots of FBS should be made for preparing fresh complete CCM (see 4.1). The FBS stock should be defrosted, aliquoted in 55 ml portions (i.e. for preparing 10% v/v final concentration in CCM) in 100-ml sterile glass bottles (or equivalent glass recipient), and stored in a freezer at -20 °C.

5. EXPERIMENTAL PROTOCOL

Note: Always use prewarmed (in 37 °C water bath) complete CCM.

5.1. Numbering of cell lines

Please refer to the scheme in ANNEX.

- The abbreviated name of a cell line is always used for identifying a cell line. Cell stocks and cultures are indicated with a 2-digit numbering: a cell line number and a passage number.
- The **cell line number** indicates how many times the cells have already been frozen and thawed in the laboratory.
- The **passage number** indicates how many times the cells with a certain cell line number have already been passed since thawing. Additionally, the total number of passages during the previous cell line numbers is mentioned between brackets.

5.2. Freezing cells

5.2.1. General notes

- Before freezing the cells, the absence of mycoplasma should be checked. Mycoplasma detection is done according to in-house procedures. If cells are mycoplasma free they can be frozen.
- Aliquots of early-passage cells (minimal 2, maximum 4 passages) should be frozen down to replace older cultures. Expand the cells (see 5.4) so that a least 10 vials can be frozen.
- Store the cryovials in different liquid nitrogen tanks if available to create a back-up of cells in case a liquid nitrogen tanks fails.

5.2.2. Freezing protocol

- Proceed as for subculturing (5.4.2) starting from a 80-90% confluent T75 flask: trypsinize cells, count them before centrifugation, and resuspend in complete CCM at a density of 1.25 x 10^6 cells/ml.
- Aliquot 800 µl (1 x 10^6 cells) to the appropriate number of cryovials.
- Add 800 µl of freezing medium.
- Transfer the vials to a freezing container and freeze the cells at -80°C overnight.
- Store the cells in liquid nitrogen for long periods of time.

5.3. Defrosting cells

- Quickly thaw frozen cells in a 37 °C water bath, until just thawed.
- Add cells to a 15 ml conical sterile tube containing 10 ml complete CCM.
- Centrifuge the cells at 150 x g for 5 min.
- Aspirate the supernatant.
- Add 2.5 ml complete CCM to a new sterile T25 flask.
- Resuspend the cells in 2.5 ml of complete CCM and add them to the T25 flask.
- Place the T25 flask in the CO_2 incubator.
- After 24 hours, check the cells under the microscope.
- Replace the complete CCM by aspirating it and gently adding fresh complete CCM.

5.4. Subculturing protocol

5.4.1. General notes

- Cell cultures are maintained in T25 or T75 cell culture flasks, in which the cells are passaged at 70-80% confluency every 3-4 days (on Monday and Friday) with medium renewal every Wednesday.

- When the T25 flask reaches 80% confluence the cells can be expanded to a T75 flask if a higher number of cells is required by the experimental procedure.
- Cells should be passaged at least 2 times before use in experiments or before freezing, and they should be passaged no more than 20 times in total.
- Mycoplasma testing of the cell cultures should be performed on a regular basis according to in-house procedures as a quality check, e.g. once every month, and before freezing cells (see 5.2).

5.4.2. Subculturing

- Check the cells under the microscope.
- Gently rinse the cells 2 times with 2.5 ml (T25) or 5 ml (T75) PBS at room temperature, aspirate and discard the PBS.
- Add 0.7 ml (T25) or 1.5 ml (T75) Trypsin-EDTA to the flask, and incubate for 3 min at 37°C in the CO_2 incubator.
- Add 3-4 ml (T25) or 6-8 ml (T75) complete CCM to the flask.
- Pipette the cells up and down 2 to 3 times to detach and suspend them, and transfer them to a centrifuge tube.
- Check under the microscope if no cells are left in the flask.
- Centrifuge the cells at 150 x g for 5 min.
- Aspirate the supernatant and resuspend the cells in 5 ml of complete CCM.
- Count the cells (dead and living) using a haemocytometer or automated cell counter.
- Absolute viability should be > 90 %.
- Transfer a volume of cell suspension equivalent to 1.5×10^5 (T25) or 4.5×10^5 cells (T75) to the flask <u>on Monday</u>. Immediately add complete CCM to a total volume of 5 ml (T25) or 15 ml (T75).
- Change the culture medium after 2 days (<u>Wednesday</u>) and subculture on <u>Friday.</u>
- If leaving the medium on the cells for 3 days from <u>Friday</u> to Monday, seed 3×10^5 cells in a total volume of 10 ml (T25) or 9×10^5 cells in a volume of 20 ml (T75) of complete CCM.

6. ACCEPTANCE criteria

Cells can be used for experiments or cell banking when the following criteria are met:
- Absolute cell viability > 90%, as determined by cell counting
- Cells have reached maximum 90% confluence

Cells are free of mycoplasma

Standard Operating Procedure for assessment of A549 cell growth rate and viability

1. Introduction

1.1. Principle

When cells undergo exponential growth the relative growth rate, and thus doubling time (i.e. the period of time required for a quantity of cells to double in number) is constant.
Knowledge of the relative growth rate of a specific cell type is useful for planning experiments, e.g. the number of cells to be seeded to obtain a particular cell concentration that is required for a study can be easily determined. It also gives you an idea of the health of your cell line.

1.2. Safety measures

- All work should be performed in a laminar flow biosafety cabinet to ensure sterile handling of cell cultures.
- All disposable materials, the remainder of the stock solutions, the content of the waste container connected to the aspirator and the remainder of the cells must be removed as toxic biologically hazardous waste.

2. EQUIPMENT and reagents

2.1. REAGENTS

- Modified Eagle Medium (MEM) with glutamax (Invitrogen, Cat. #41090093)
- Fetal Bovine Serum (FBS), non-heat activated
- (Penicillin/streptomycin) (Invitrogen Cat.# 15070063)
- 0.05% Trypsin-EDTA (Invitrogen Cat.# 25300054)
- Trypan blue stain, 0.4% in phosphate buffered saline (depends on counting method)

2.2. EQUIPMENT

- Micropipettors + sterile tips
- CO_2 incubator[1] at 37±2 °C, 5±0.5 % CO_2, 95 % relative humidity
- Centrifuge
- Aspirator + waste container
- Light microscope
- Refrigerator (4 °C) and Freezer (-20 °C)
- Cell counter[2] (e.g. Countess Cell Counter, Invitrogen)
- Water bath (37±2 °C)
- 15 and 50 ml sterile conical polystyrene tubes
- 1.5 ml Eppendorf tubes
- 24-well plates
- Vortex

[1]Verify that the incubator has been recently calibrated for temperature, humidity and CO_2 concentration.

[2] If you are using an automated cell counter, make sure that is has been calibrated/verified for the cell type you are using.

3. SOLUTIONS

3.1. Complete cell culture medium (CCM)

Composition:
MEM with glutamax
(100 U/ml Penicillin)
(100 μg/ml streptomycin)
10% FBS

Note: Complete CCM needs to be stored at 4°C.
Note: The use of antibiotics-free CCM is preferred for cell culture. Only during exposure experiments antibiotics can be used.

4. EXPERIMENTAL PROTOCOL

4.1. Plating cells

- Cell cultures are maintained as described in the SOP on A549 CELL CULTURING.
- Use one 24-well plate for each time period you want to measure (e.g. 24, 48 and 72 hours), the plate layout is shown in figure 1. At least two, but preferably three time points are needed to determine the cell growth rate accurately.
- The cells should be collected as specified in the cell culture SOP for the used cell type, counted and checked for at least 90% viability, and then resuspended in complete CCM at 0.5 x10^5 cells/mL.
- The 24-well plate is put on a wet tissue before plating to prevent that cells will clump in the center or periphery of the wells (edge effect).
- Plate your cells at 0.5 x10^5 cells in 1 mL per well with 8 replicates per time period using a single channel pipettor. The wells at the periphery are filled up with medium (or sterile PBS).
- Leave the cells to stand at room temperature under the sterile flow for 15 min and then gently transfer the plate to a humidified incubator at 37 °C with 5% CO_2 so that cells do not clump in the center or periphery of the wells.

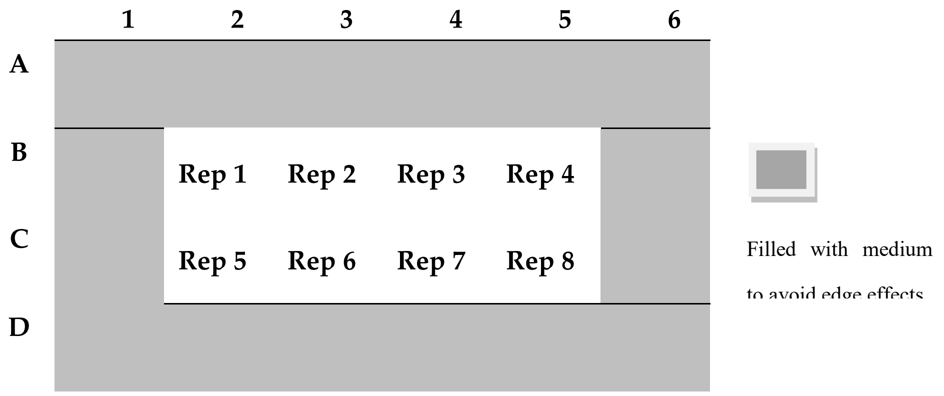

Figure 1. Cell Plate Layout. Cells are seeded at 0.5 x10^5 cells/well in each of the open wells. The grey wells are filled with medium to avoid edge effects. Cells in a single well should be seeded with a single channel pipetting step.

4.2. Determination of cell number and viability

- At each time point, remove one plate from the incubator.
- Check the cells under the microscope and record the morphology, apparent health and percentage confluence.
- Harvest the cells of each well as specified in the cell culture SOP for the used cell type, use 200 µl Trypsin-EDTA per well for adherent cells. When cells are loose, add 400 µl of complete medium to each well and collect the cells of each well in a separate centrifuge tube.
- Rinse the wells by adding another 400 µl of complete CCM to each well and pipetting gently, and collect the remaining cells in the corresponding tube.
- The resulting cell suspension may be used directly for counting your cells. If the cell suspension needs to be more concentrated for your counting method, centrifuge the tubes (150 x g, 5 min), discard the supernatant, add 100 µl of complete CCM to each tube, and resuspend the pellet by pipetting.
- Count the number of live and dead cells per replicate tube, either manually using a hematocytometer under the microscope or with an automated cell counter.

5. Data Analysis

5.1. Cell counts and viability

Note the final numbers of live/dead cells (absolute numbers or cells per ml) and/or total cell count, taking into account any dilution or concentration steps applied in 4.2. Calculate the total cell count and percent viability (% of live cells of the total number of cells).

5.2. Relative growth rate

Determine the relative growth rate of your cell type by using the growth duration times and the live cell counts (i.e. absolute number of live cells or cells/mL), and report the results. Cell counts that correspond to growth saturation (for adherent cells when confluence is over 100%) should not be included.

6. Acceptance criteria

- The absolute viability of the cell culture used in the test is higher than 90%.
- When comparing the relative growth rate with data from literature, guidelines or as provided by the cell supplier, the standard deviation should not be more than 20 %.The coëfficiënt of variation (CV) of the average cell counts and cell viability determinations of technical replica measurements is lower than 30%.

Calculation template for assessment of A549 cell growth rate and viability

Research Infrastructure QualityNano	Assessment of cell growth rate and viability<br>TEMPLATE: RELATIVE GROWTH RATE & VIABILITY CALCULATION	PAGE: 1 of 1

Laboratory:
Technician:
Date:
Cell type: A549

	Live Cell Counts*					Dead Cell Counts*				Total Cell Counts$		
**Rep #**	**0**	**24**	**48**	**72**	**Rep #**	**24**	**48**	**72**	**Rep #**	**24**	**48**	**74**
1	5.00E+04	1.07E+05	2.04E+05	3.99E+05	1	8.89E+03	1.11E+03	1.89E+04	1	1.16E+05	2.06E+05	4.18E+05
2	5.00E+04	1.09E+05	1.82E+05	3.26E+05	2	2.22E+03	3.33E+03	1.70E+04	2	1.11E+05	1.86E+05	3.43E+05
3	5.00E+04	1.37E+05	1.69E+05	3.46E+05	3	2.22E+03	3.33E+03	1.33E+04	3	1.39E+05	1.72E+05	3.59E+05
4	5.00E+04	1.18E+05	2.10E+05	3.22E+05	4	3.33E+03	0.00E+00	2.22E+04	4	1.21E+05	2.10E+05	3.44E+05
5	5.00E+04	8.22E+04	1.64E+05	4.02E+05	5	4.44E+03	4.44E+03	2.00E+04	5	8.67E+04	1.69E+05	4.22E+05
6	5.00E+04	1.13E+05	1.96E+05	3.78E+05	6	4.44E+03	4.44E+03	3.00E+04	6	1.18E+05	2.00E+05	4.08E+05
7	5.00E+04	9.78E+04	1.96E+05	4.18E+05	7	7.78E+03	4.44E+03	2.11E+04	7	1.06E+05	2.00E+05	4.39E+05
8	5.00E+04	8.11E+04	1.72E+05	3.32E+05	8	2.22E+03	3.33E+03	1.44E+04	8	8.33E+04	1.76E+05	3.47E+05
**Average**	**5.00E+04**	**1.06E+05**	**1.87E+05**	**3.65E+05**	**Average**	**4.44E+03**	**3.06E+03**	**1.96E+04**	**Average**	**1.10E+05**	**1.90E+05**	**3.85E+05**
**STDV**	**0.00E+00**	**1.85E+04**	**1.71E+04**	**3.83E+04**	**STDV**	**2.59E+03**	**1.65E+03**	**5.22E+03**	**STDV**	**1.82E+04**	**1.62E+04**	**4.04E+04**
**CV (%)**	**0.0**	**17.5**	**9.2**	**10.5**	**CV (%)**	**58.2**	**54.1**	**26.6**	**CV (%)**	**16.6**	**8.5**	**10.5**

*Exact growth duration times, and absolute live cell numbers or cells per mL should be entered here; leave out cell counts at saturation (i.e. confluence > 100%)
$Total cell counts should be entered here or calculated from live+dead cell counts.

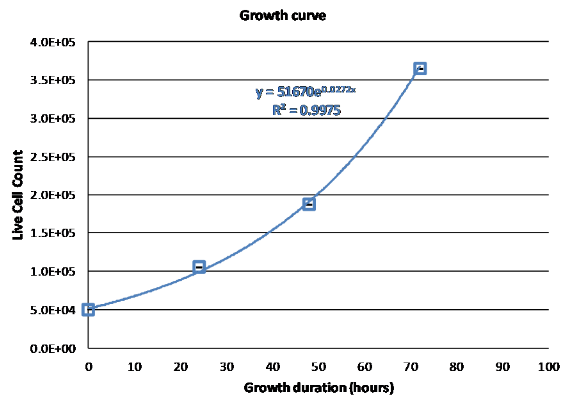

	Cell Viability (%)		
**Rep #**	**24**	**48**	**72**
1	92.3	99.5	95.5
2	98.0	98.2	95.0
3	98.4	98.1	96.3
4	97.2	100.0	93.5
5	94.9	97.4	95.3
6	96.2	97.8	92.6
7	92.6	97.8	95.2
8	97.3	98.1	95.8
**Average**	**95.9**	**98.3**	**94.9**
**STDV**	**2.4**	**0.9**	**1.2**
**CV (%)**	**2.5**	**0.9**	**1.3**

Relative Growth Rate:

**DATA FROM EQUATION:**	prefactor of power =	0.0272
(to be filled in by operator)		
**CALCULATION:**	**Doubling time =**	**25 hours**
	**Relative growth rate =**	**0.0272 doublings per hour**

Standard Operating Procedure for the MTS cytotoxicity assay

1. Introduction

1.1. Principle

The CellTiter 96® AQueous One Solution Cell Proliferation Assay is a colorimetric method for determining the number of viable cells in proliferation or cytotoxicity assays.

The CellTiter 96® AQueous One Solution Reagent contains a novel tetrazolium compound [3-(4,5-dimethylthiazol-2-yl)-5-(3-carboxymethoxyphenyl)-2-(4-sulfophenyl)-2H-tetrazolium, inner salt; MTS] and an electron coupling reagent (phenazine ethosulfate; PES). PES has enhanced chemical stability, which allows it to be combined with MTS to form a stable solution.

The MTS tetrazolium compound (Owen's reagent) is bioreduced by mitochondria of viable cells into a colored formazan product that is soluble in tissue culture medium. This conversion is presumably accomplished by NADPH or NADH produced by dehydrogenase enzymes in metabolically active cells.

Assays are performed by adding a small amount of the CellTiter 96® AQueous One Solution Reagent directly to culture wells, incubating for 1–4 hours and then recording the absorbance at 490 nm with a 96-well plate reader.

1.2. Application area

The MTS assay is used to determine the possible effect of a test item on viable cell proliferation. Test items should not interfere with the reagent (e.g. by reduction) or read-out method (absorbance at 490 nm). Adherent cell types that are free of mycoplasma can be used in the assay, but also suspension cells can be tested with inclusion of adaptations to the below described protocol.

1.3. Safety measures

- In-house procedures for safe handling of nanomaterials and other potentially hazardous compounds should be followed.
- All work should be performed in a laminar flow Biosafety cabinet to ensure sterile handling of cell cultures.

2. EQUIPMENT and reagents

2.1. REAGENTS

- Modified Eagle Medium (MEM) with glutamax (Invitrogen, Cat. # 41090093)
- MilliQ sterile water (18.2 mΩ resistivity at 25°C)
- Fetal Bovine Serum (FBS), non-heat inactivated
- Penicillin/streptomycin (Invitrogen Cat.# 15070063)
- CellTiter 96® AQueous One Solution Reagent (Promega Cat. G3580/G3581/G3582)
- Staurosporine (MW: 466.53), 1 mg (Proteinkinase.de, Cat.# PKI-STSP-001)
- DMSO, cell biology grade
- 50 nm Polystyrene-amine nanoparticles (Bangs Laboratories, Cat. #PA02N) diluted in water to 10 mg/ml
- 40 nm fluorescently labelled Polystyrene-carboxylate nanoparticles (Molecular Probes, Cat. #F8795) diluted in water to 10 mg/ml

2.2. EQUIPMENT

- 50 ml sterile conical polystyrene tubes
- 1.5 ml sterile cryotubes
- 2 and 15 ml sterile polypropylene tubes
- Sterile V-shaped multichannel pipette reservoir
- Flat-bottom transparent 96 multi-well plates
- Round-bottom 96 multi-well plates for dosing plate preparation
- Microtiter plate reader with 490 nm filter (possible range 450-540 nm)[1]
- Micropipettor + sterile tips
- Multichannel pipettors[2], with 100 and 200 µl volume/pipette
- Biohazard
- CO_2 incubator[3] at 37±2 °C, 5±0.5 % CO_2, 95 % relative humidity
- Centrifuge
- Aspirator + waste container
- Light microscope
- Refrigerator(4 °C) and Freezer (-20 °C)
- Cell counter[4] (e.g. Countess Cell Counter, Invitrogen)
- Water bath (37±2 °C)
- Vortex

[1]To verify the performance of the microtiter plate reader with respect to minimal well-to-well variability, an absorbance measurement reference plate (delivered by the supplier of the reader) can be run. If this is not available a 96-well plate with each well filled with identical volumes of MTS assay reagent or a similar absorbing solution (e.g. CCM + 10 % FBS) in each well can be run. Pour 10 mL of absorbing solution into a V-shaped reservoir. Using a multichannel pipettor with 6 pipet tips, add 200 µL to each column 2-10 (rows B to G). Each well of the plate should be read and the raw absorbance values added to the spread sheet. The absorbance measurements from each well should show no deviation larger than 5% CV from the overall mean. In case of outliers, a new plate should be prepared and the procedure is repeated. If the problem persists, verify your pipettor, check your pipetting technique, try another plate reader or contact your supplier.

[2]Multichannel pipettors have to be verified every two months. Verify two channels per verification time. Verify two other channels in the next verification round. E.g. channels 1 and 8 are verified in January, channels 2 and 6 are verified in March.

[3]Verify that the incubator has been recently calibrated for temperature, humidity and CO2 concentration.

[4]In case an automated cell counting system is used, make sure its performance has been verified according to the manufacturer's recommendations before measurement.

3. MEDIA, SOLUTIONS AND DISPERSIONS

3.1. Complete cell culture medium (CCM)

<u>Composition:</u>

MEM with glutamax
(100 U/ml Penicillin)
(100 µg/ml streptomycin)
10% FCS

Note: Complete CCM needs to be stored at 4°C.

Note: The use of antibiotics-free CCM is preferred for cell culture and preparation of the cell plate (section 4.1). However, addition of antibiotics to the medium is recommended for preparation of the MTS reagent (section 3.2), NP and chemical solutions, and the dosing plate (section 3.3).

3.2. MTS reagent

- Thaw the CellTiter 96® AQueous One Solution Reagent. It should take approximately 90 minutes at room temperature, or 10 minutes in a water bath at 37 °C, to completely thaw the 20 ml size.
- Prepare 10.2 mL of MTS reagent for each plate by mixing 1.7 mL of AQueous reagent with 8.5 mL of complete CCM (containing antibiotics) in a 15 ml polypropylene tube. Make sure no precipitate is present in the solution.

Note: For long-term storage, store the CellTiter 96® AQueous One Solution Reagent at –20°C, protected from light. See the expiration date on the Product Information Label.

3.3. Test items

3.3.1. Preparation of NP dispersions in CCM

- This procedure should be performed in a biohazard or in a cleaned area that minimizes contamination of the CCM with bacteria or fungi.
- NP stock suspensions should be stored at 4°C. Suspensions of 10 mg/ml NP remain stable for at least 6 months under these conditions.
- All NP dispersions are prepared freshly from stock suspensions following the below dispersion protocol.
- Variability due to NP preparation is estimated by preparing 3 separate NP dispersions (see below) each representing a separate technical replicate (columns 8, 9, and 10, respectively, in fig. 1).
- Pipette 1980 µl of complete CCM into three separate sterile 2 ml polypropylene tubes.
- Vortex the NPs suspensions for 30 sec. on a benchtop vortex at full speed.
- Immediately add 20 µl of the received suspension to each tube to reach a final concentration of 100 µg/ml.
- Immediately vortex the dilution briefly for 30 sec. using a benchtop vortex at full speed.
- These triplicate NP dilutions are immediately used to prepare the dosing plate for cell exposure (steps 3.3.3 and 4).

Note: For parallel NP size characterization using DLS (or alternatively CPS or NTA) an additional set of triplicate NP dispersions in CCM, and a set in water as a reference is prepared according to the same principle as described above. When using NTA or CPS the volume of the NP solutions may need to be adapted.

3.3.2. Preparation of positive control chemical solution

Staurosporine will be used as positive control chemical which is known to induce apoptotic processes. Different doses of the chemical will be applied to serve as an internal control of the assay's and user's performance. In A549 cells a dose of 1 µM Staurosporine will induce a clear cytotoxic response after 24 hours exposure, and is prepared as follows:

Stock solution:

- Dissolve 1 mg Staurosporine (lyophylized powder) by adding 214 µl DMSO to the original vial, and mixing on a vortex.

- Dilute the resuspended Staurosporine immediately further to 2140 µl in DMSO to obtain a 1 mM stock solution. This is done by transferring the resuspended product to a fresh sterile tube, and adding the DMSO while rinsing the original vial to make sure all is collected. Mix well again on a vortex.
- Aliquot the 1 mM stock solution in portions of 50 µl in sterile cryotubes and store at -80°C without loss of activity for at least 6 months. A fresh tube is thawn for each separate experiment as repeated thaw-freeze cycles should be avoided to maintain the stability of Staurosporine.

Working solution:
Note: Please avoid pipetting volumes below 10 µl to minimize pipetting errors.

- As complete CCM (with antibiotics added) containing 1% H_2O is used as vehicle in dissolving the NP, Staurosporine working solutions are prepared in the same medium to ensure that each well in the assay plate has identical CCM. Complete CCM containing 1% H_2O is prepared in a 50 mL conical tube, by adding 49.5 mL of complete CCM and 0.5 mL of sterile H_2O. Mix by inversion.
- Working solutions of 1 µM Staurosporine are prepared freshly before use by adding 450 µl complete CCM containing 1% H_2O directly to a freshly thawn tube of the stock solution (1 mM) to obtain an intermediate solution of 100 µM.
- Separate Staurosporine working solutions of 1 µM are prepared as three independent technical replicates (columns 3, 4, and 5, respectively, in fig. 1) to assess variability in preparation by diluting 20 µl of the 100 µM intermediate solution in 1980 µl complete CCM with 1% H_2O in separate 2 ml polypropylene tubes.

3.3.2. Preparation of the dosing plate

- The dosing plate layout, containing three technical replicate columns of serial dilutions of the positive control chemical and NP, is shown in Figure 1. Chemical and NP dilutions are prepared in a round-bottom 96 multi-well plate immediately before exposure of the cells.
- To ensure that each well in the assay plate has identical CCM, it is important to use complete CCM (with antibiotics added) containing 1% H_2O for preparation of the dosing plate. See 3.3.2 for preparation.
- Transfer 250 µL (excess) of complete CCM containing 1% H_2O into the wells in columns 6 and 7 (no treatment), and transfer 250 µL of the highest test doses 1 µM Staurosporine and 100 µg/ml NP of one of the three replicate dilutions series into the wells of columns 2 and 11, respectively.
- Transfer a volume of complete CCM into the remaining columns 3-5 and 8-10 as specified in table 1 using a single channel pipettor. The same tips can be used.
- The chemical and NP dosing columns (3-5 and 8-10, respectively) should be prepared by serial dilution starting from the highest test dose (row G) as described in Table 1 using a multichannel pipettor with 3 pipet tips.
- Put the dosing plate in the incubator at 37°C until use.

Table 1. Preparation of chemical (columns 3-5) and NP (columns 8-10) serial dilutions on Dosing Plate (always make an excess).

Row Number	*Complete CCM with 1% H_2O (µL) in Columns 3-5*	*Positive Control chemical dilutions in columns 3-5 (µL)*	*Complete CCM with 1% H_2O (µL) in Columns 8-10*	*NP dilutions in columns 8-10 (µL)*
B	*250*	*0*	*250*	*0*
C	*125*	*125 of row D*	*225*	*25 of row D*
D	*125*	*125 of row E*	*150*	*100 of row E*
E	*125*	*125 of row F*	*125*	*125 of row F*
F	*125*	*125 of row G*	*125*	*125 of row G*
G	*0*	*250 (1 µM, see 3.3.2)*	*0*	*250 (100 µg/ml, see 3.3.1)*

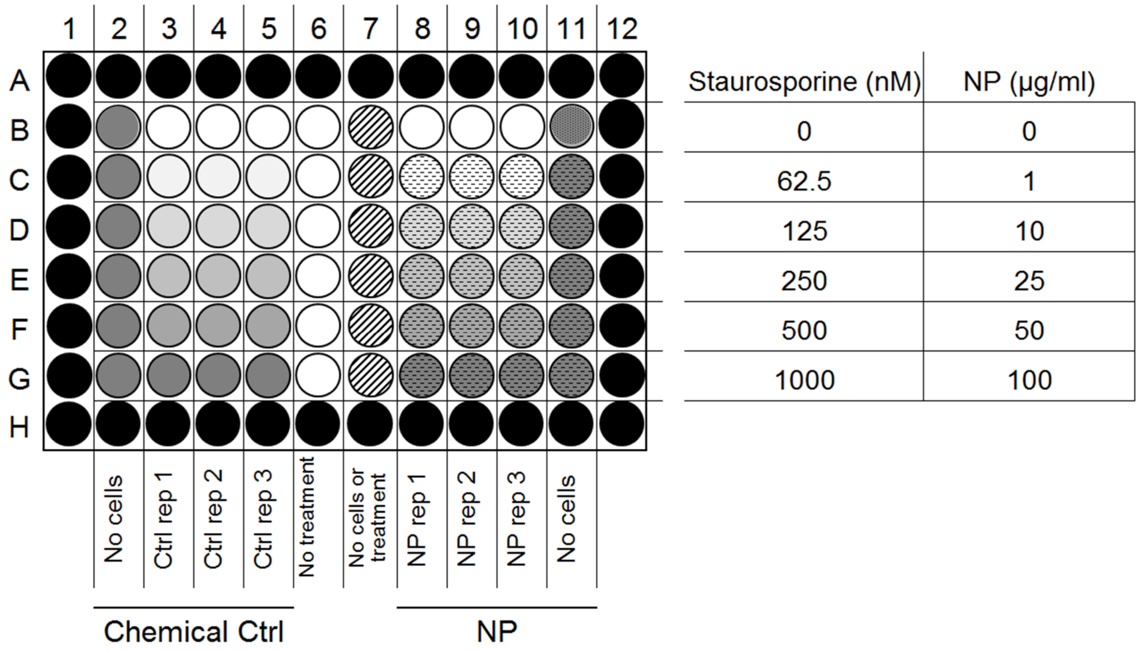

Figure 1. Dosing Plate Layout. The blue and hatched wells contain complete CCM +1% H_2O. Columns 3-5 and columns 8-10 contain chemical control and NP doses, respectively. Columns 2 and 11 contain the highest test dose of chemical control and NP, respectively. See Table 1 for volumes. The contents of these wells are transferred per row to the cell plate from the lower to the higher dose with a multichannel pipettor.

4. EXPERIMENTAL PROTOCOL

4.1. Preparation of the Cell Plate

- Cell cultures are maintained as described in the SOP on A549 CELL CULTURING.
- The cell plate layout is shown in Figure 2, this is prepared in a flat-bottom 96-well plate.
- Complete CCM with no cells (200 µL) is transferred into each well in columns 2, 7 and 11, as well as in the wells of the periphery (black wells) to avoid edge effects.
- Approximately 500,000 live cells in 10 ml are needed for a single plate.

- The cells should be collected as specified in the cell culture SOP for the used cell type, counted and then resuspended in complete CCM at ~5 x10^4 live cells/mL.
- The cells are then added to a V-shaped multichannel pipette reservoir and a multichannel pipettor with 6 pipet tips is used to transfer 200 μL of cell suspension into a single column of the flat-bottom 96-well plate using a forward pipetting technique. To optimize homogeneity in preparing the cell plate, it is important to use separate pipetting actions in the V-shaped reservoir to mix the cell suspension between each plating step. This minimizes settling of the cells on the reservoir bottom.
- Columns 3-6 and 8-10 are seeded with cells. To prevent that cells will clump in the center or periphery of the wells due to edge effects, the 96-well plate is put on a wet tissue while plating, and cells are left to stand at room temperature under the sterile flow for 15 min. The necessity of these steps should be assessed for each studied cell type.
- Gently transfer the plate to a humidified incubator at 37 °C with 5% CO_2.

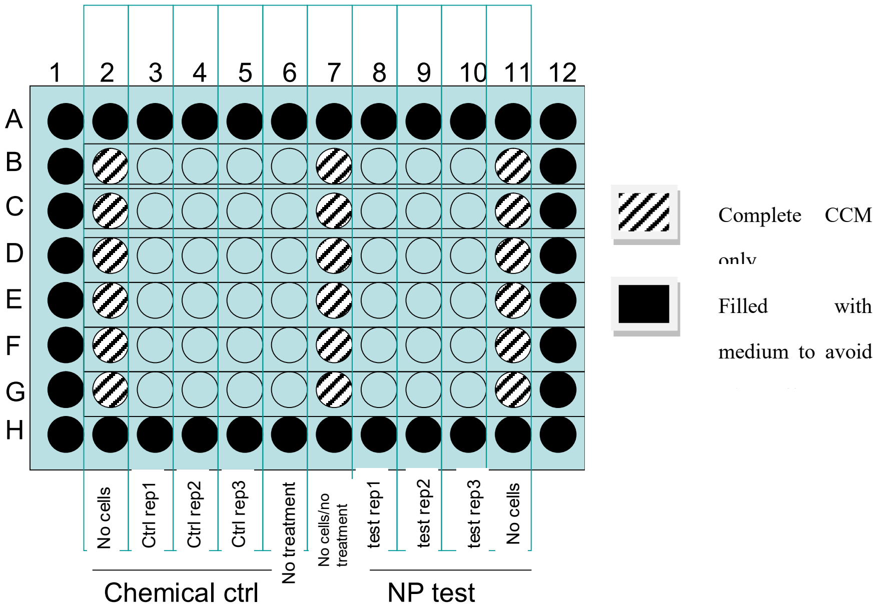

Figure 2. Cell Plate Layout. Cells are seeded at 1x10^4 cells/well in each of the blue wells. The striped wells contain complete CCM only and the black wells are filled with medium to avoid edge effects. All cells in a single column should be seeded with a single multichannel pipetting step.

4.2. Exposure to test item

- After the cell plate has been incubated for 24 h, check the plate under the microscope (no contamination, cell attachment and density,...). The CCM is then carefully removed from each well with a multichannel pipettor positioned at the bottom edge of the well (take care not to disturb the attached cell layer), except for the peripheral wells.
- The contents of each row in the dosing plate (Figure 1) are then gently mixed by several pipetting actions and carefully added to the cell plate using a single forward pipetting action with a multichannel pipettor equipped with 10 pipet tips set at 100 μL. Chemical and NP doses are applied onto the cells from the lower to the higher concentration so that the same tips can be used without affecting the concentration.

- Check the cells again under the microscope to see if they are still well attached.
- The plates are then transferred to the incubator for 24 h.

4.3. MTS assay

- Check the individual wells of the cell plate at the end of the exposure period under a microscope.
- Prepare 10.2 mL MTS Reagent for each plate (see 3.2).
- Remove the dosing treatments and non-adhered unhealthy cells using a multichannel pipettor from the side of the well while preventing healthy cells to get loose.
- Using a multichannel pipettor with 6 pipet tips, transfer 150 µL of the MTS reagent into each column of the cell plate. Do not use the expel step in the pipetting procedure to prevent the formation of air bubbles (reverse pipetting).
- Check the wells for the presence of air bubbles and remove them using a syringe needle (air bubbles in a well can significantly affect the absorbance measurement with a plate reader. It is important to note which wells may have air bubble issues before placing the plate on the plate reader. The information should be used during the validation of the well absorbance measurements).
- Incubate the plate at 37 °C for 1 hour in a humidified, 5% CO_2 atmosphere.
- Formazan absorbance is measured at 490 nm using a microtiter plate reader. Set the plate reader so that the plate is shaken for 10 sec to homogenize possible brownish precipitate before raw absorbance measurements are recorded for every well in the plate. The amount of coloured product formed is proportional to the number of live cells in culture. If a treatment causes cell proliferation, viability will appear to increase.
- After the first reading, transfer 100 µL of the MTS reagent of each well into a new transparent 96-well flat-bottom plate, make sure no air bubbles are present in the wells (reverse pipetting), and read again the absorbance at 490 nm.

5. Data analysis

The raw data from every well in the plate should be recorded for full data and variance analysis. Data analysis is performed based on the following procedure:

- The average background level (column 7 = no cells/no treatment) should be subtracted from each well to obtain blank-corrected absorbance values (Abs_c).
- The resulting absorbance from each dosing well in a technical replicate is then normalized to the average blank-corrected absorbance value of the no-treatment wells (column 6). The resulting values are the fraction of cells that remain after dose treatment, which can be expressed as percentage cell survival.

$$\textbf{\% cell survival} = \frac{\textbf{Individual } \mathbf{Abs_c} \textbf{ treated cells x 100\%}}{\textbf{Average } \mathbf{Abs_c} \textbf{ non-treated cells}}$$

- After normalization, the values from a single dose in the 3 technical replicates can be averaged and standard deviations can be determined.

An IC_{30} or IC_{50} value can be estimated from the dataset by simple extrapolation, non-linear regression analysis using GraphPad Prism or other software applications.

6. Acceptance criteria

The test system is considered suitable for application with the test items when the following criterion is met:

- No interference of the test items (chemical control or NP) with MTS is observed at the highest test doses. Therefore, the individual raw absorbance values of the chemical control blank (column 2) and NP test blank (column 11) should show less than 15% difference as compared to the average absorbance value of the blank wells (column 7).

The test run is accepted when the following criteria are met:

- The absolute viability of the cell culture used in the test is higher than 90%, as determined by cell counting.
- The cells in no-treatment wells (column 6) do not show obvious cytotoxicity at the end of the exposure period, as observed by microscopic observation.
- The coëfficiënt of variation of the average absorbance value of the blank wells (no-cell/no-treatment, column 7) is lower than 15%.
- The average absorbance values of technical replicate measurements of cells treated with zero dose (row B) should not deviate more than 15% from the average absorbance of non-treated cells (column 6).
- The coëfficiënt of variation of the average absorbance value Abs_c of technical replica measurements is lower than 30% when individual $Abs_c > 0.1$.
- At least one of the applied doses of the positive control chemical gives rise to <70% cell survival as compared to non-treated cells (for which the average cell survival is set to 100%).

Calculation template for the MTS cytotoxicity assay

Research Infrastructure QualityNano	MTS cytotoxicity assay<br>TEMPLATE: CELL SURVIVAL CALCULATION	PAGE: 1 of 1

Laboratory:
Technician:
Date:
Cell type: A549 **Seeding density:** 10000 cells/well

Paste Raw Absorbance Data:

	1	2	3	4	5	6	7	8	9	10	11	12
		Ctrl_BLANK ↓	Ctrl_Rep 1	Ctrl_Rep 2	Ctrl_Rep 3	No treatment	No Tr_BLANK ↓	Test_Rep 1	Test_Rep 2	Test_Rep 3	Test_BLANK ↓	
A												
B		0.120	1.127	1.216	1.224	1.247	0.118	1.267	1.289	1.304	0.120	
C		0.119	1.087	1.168	1.177	1.258	0.122	1.302	1.300	1.288	0.121	
D		0.118	1.104	1.146	1.137	1.253	0.118	1.260	1.270	1.281	0.120	
E		0.119	1.009	1.042	1.019	1.216	0.118	1.204	1.243	1.248	0.122	
F		0.119	0.714	0.749	0.773	1.265	0.118	1.153	1.148	1.132	0.128	
G		0.120	0.696	0.739	0.747	1.292	0.119	0.349	0.392	0.381	0.131	
H												

Results per well

		Ctrl_BLANK ↓	Ctrl_Rep 1	Ctrl_Rep 2	Ctrl_Rep 3	No treatment	No Tr_BLANK ↓	Test_Rep 1	Test_Rep 2	Test_Rep 3	Test_BLANK ↓	
A												
B		0.120	1.127	1.216	1.224	1.247	0.118	1.267	1.289	1.304	0.120	
C		0.119	1.087	1.168	1.177	1.258	0.122	1.302	1.300	1.288	0.121	
D		0.118	1.104	1.146	1.137	1.253	0.118	1.260	1.270	1.281	0.120	
E		0.119	1.009	1.042	1.019	1.216	0.118	1.204	1.243	1.248	0.122	
F		0.119	0.714	0.749	0.773	1.265	0.118	1.153	1.148	1.132	0.128	
G		0.120	0.696	0.739	0.747	1.292	0.119	0.349	0.392	0.381	0.131	
H												
Average		0.119					0.119				0.123	
STDV		0.001					0.002				0.003	
CV (%)		0.5					1.7				2.8	

Averaged blank

	Average	STDV	CV (%)
No Tr_BLANK	0.119	0.002	1.5

Results accepted if CV<30%

Blank-corrected Absorbance Values (Abs$_c$)

Dose (nM)	Ctrl_Rep 1	Ctrl_Rep 2	Ctrl_Rep 3	Crtl_Staurosporine Average	STDV	CV (%)
0	1.008	1.097	1.105	1.070	0.054	5.0
62.5	0.968	1.049	1.058	1.025	0.049	4.8
125	0.985	1.027	1.018	1.010	0.022	2.2
250	0.891	0.923	0.900	0.904	0.017	1.9
500	0.595	0.630	0.655	0.626	0.030	4.8
1000	0.577	0.620	0.628	0.608	0.028	4.5

	No treatment
	1.128
	1.139
	1.134
	1.097
	1.146
	1.173
Average	1.136
STDV	0.025
CV (%)	2.2

Dose (µg/ml)	Test_Rep 1	Test_Rep 2	Test_Rep 3	Test_Nanomaterial Average	STDV	CV (%)
0	1.148	1.170	1.185	1.168	0.019	1.6
1	1.183	1.181	1.169	1.178	0.007	0.6
10	1.141	1.152	1.162	1.151	0.010	0.9
25	1.085	1.124	1.129	1.113	0.024	2.2
50	1.034	1.029	1.013	1.026	0.011	1.1
100	0.230	0.273	0.262	0.255	0.023	8.8

Results accepted if CV < 30% when Abs$_c$ > 0.1, or if Abs$_c$ < 0.1

Cell survival (%)

Dose (nM)	Ctrl_Rep 1	Ctrl_Rep 2	Ctrl_Rep 3	Crtl_Staurosporine Average	STDV
0	88.7	96.6	97.2	94.2	4.7
62.5	85.2	92.3	93.1	90.2	4.3
125	86.7	90.4	89.6	88.9	1.9
250	78.4	81.3	79.2	79.6	1.5
500	52.4	55.4	57.6	55.1	2.6
1000	50.8	54.6	55.3	53.5	2.4
X				Y	

Dose (µg/ml)	Test_Rep 1	Test_Rep 2	Test_Rep 3	Test_Nanomaterial Average	STDV
0	101.0	103.0	104.3	102.8	1.6
1	104.1	103.9	102.9	103.6	0.7
10	100.4	101.4	102.2	101.3	0.9
25	95.5	98.9	99.4	97.9	2.1
50	91.0	90.6	89.2	90.3	1.0
100	20.2	24.1	23.1	22.5	2.0
X				Y	

IC30-IC50 determination by interpolation:

Ctrl_Staurosporine	
Cell survival: 70	
Y1<70%:	55.1
Y2>70%:	79.6
X1 (at Y1):	500.0
X2 (at Y2):	250.0
IC30 (nM):	348.0

Test_Nanomaterial	
Cell survival: 50	
Y1<50%:	22.5
Y2>50%:	90.3
X1 (at Y1):	100.0
X2 (at Y2):	50.0
IC50 (µg/ml):	79.7

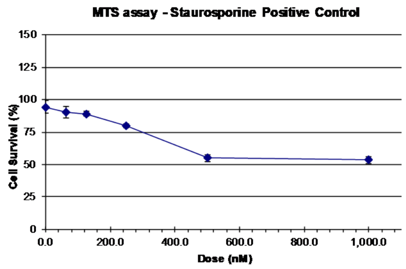

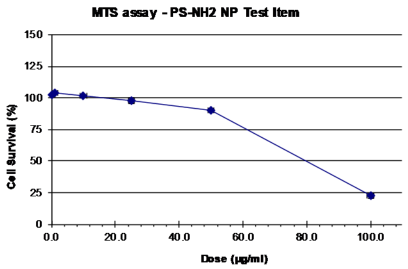

Supplement: Supplementary file 1 [file nanomaterials-10-01430-s001.pdf]
